# RNAi suppression of xylan synthase genes in wheat starchy endosperm

**DOI:** 10.1371/journal.pone.0256350

**Published:** 2021-08-19

**Authors:** Mark D. Wilkinson, Ondrej Kosik, Kirstie Halsey, Hannah Walpole, Jessica Evans, Abigail J. Wood, Jane L. Ward, Rowan A. C. Mitchell, Alison Lovegrove, Peter R. Shewry

**Affiliations:** 1 Plant Science Department, Rothamsted Research, Harpenden, United Kingdom; 2 Computational and Analytical Sciences, Rothamsted Research, Harpenden, United Kingdom; Institute of Genetics and Developmental Biology Chinese Academy of Sciences, CHINA

## Abstract

The xylan backbone of arabinoxylan (AX), the major cell wall polysaccharide in the wheat starchy endosperm, is synthesised by xylan synthase which is a complex of three subunits encoded by the GT43_1, GT43_2 and GT47_2 genes. RNAi knock-down of either GT43_1 or all three genes (triple lines) resulted in decreased AX measured by digestion with endoxylanase (to 33 and 34.9% of the controls) and by monosaccharide analysis (to 45.9% and 47.4% of the controls) with greater effects on the amount of water-extractable AX (to 20.6 and 19.9% of the controls). Both sets of RNAi lines also had greater decreases in the amounts of substituted oligosaccharides released by digestion of AX with endoxylanase than in fragments derived only from the xylan backbone. Although the GT43_1 and triple lines had similar effects on AX they did differ in their contents of soluble sugars (increased in triple only) and on grain size (decreased in triple only). Both sets of transgenic lines had decreased grain hardness, indicating effects on cell wall mechanics. These results, and previously published studies of RNAi suppression of GT43_2 and GT47_2 and of a triple mutant of GT43_2, are consistent with the model of xylan synthase comprising three subunits one of which (GT47_2) is responsible for catalysis with the other two subunits being required for correct functioning but indicate that separate xylan synthase complexes may be responsible for the synthesis of populations of AX which differ in their structure and solubility.

## 1 Introduction

Cereal grains and products are the major sources of dietary fibre in the human diet. Furthermore grain fibre, and particularly the fibre present in whole grain, has well-established health benefits in reducing the risk of chronic diseases and certain forms of cancer [1]. However, whereas whole wheat grains contain between about 10% and 15% fibre (dry weight basis) [2] most foods are made from white flour which is derived from the starchy endosperm of the grain and has a much lower fibre content (up to about 5%) [3]. Hence, increasing the fibre content of white flour could have significant benefits for human health.

The major dietary fibre components in plants are cell wall polysaccharides, with the major dietary fibre component of wheat endosperm being arabinoxylan (AX). AX accounts for about 70% of the total cell wall polysaccharides, with other components being (1→3) (1→4) β-glucans (20%), glucomannan (2–7%) and cellulose (2–4%) [4]. However, a recent study suggested that the proportions of β-glucans and cellulose may be reversed, with about 20% cellulose and 5–6% β-glucan [5].

The AX present in white flour has a simple structure, consisting of a backbone of β-D-xylopyranosyl (xylose) residues linked through (1,4) linkages. Some xylose residues are substituted with α-L-arabinofuranosyl (arabinose) residues at either one position (position O-3) or at two positions (positions O-2 and O-3) while some arabinofuranosyl residues at position O-3 of the xylose residues may themselves be substituted with ferulic acid at the O-5 position. Ferulates present on adjacent AX chains can also form cross-links, by oxidation to give dehydrodimers (diferulates). Arabinosylation and feruloylation are important parameters as they affect the physio-chemical properties (notably solubility and viscosity) of AX and therefore the end use properties. These not only include the quality for food processing and human health but also the quality for distilling, bioethanol production and livestock feeds [6]. In particular, whereas xylan is insoluble due to the presence of inter-chain hydrogen bonds, substitution with arabinose increases the solubility by hindering their formation. However, the introduction of cross-links results in insoluble polymers which can form hydrated gels. Only about 25–35% of AX in endosperm is water soluble.

Cell wall polysaccharides are synthesized by glycosyltransferases (GT), which are one of the largest superfamilies of enzymes found in plants with most being located in the Golgi apparatus [7]. About 124 GTs have been shown to be associated with cell wall synthesis in the wheat starchy endosperm with glycosyltransferases for xylan backbone synthesis (GT47_2, GT43_2 and GT43_1) and the arabinosylation of xylan (GT61_1) being the most highly expressed [8–10].

Xylan synthase is considered to be a complex of three subunits encoded by IRX14, IRX9 (both family GT43) and IRX10 (GT47) genes and elimination of any one of these components completely disables xylan synthesis [11, 12]. IRX10 appears to be responsible for catalysis but IRX9 and IRX14 are both required for correct localisation of the complex in the Golgi [10]. The IRX14 component may play a key role in assembly of the complex in wheat [13] and its knock-out reduces xylan in primary cell walls of *Brachypodium* with profound consequences for growth [14].

In wheat endosperm, the genes that are most likely to encode the components of xylan synthase are GT43_1 (IRX14 homologue), GT43_2 (known to be a functional IRX9; [15]) and GT47_2 (IRX10 homologue), as these are the most highly expressed IRX14, IRX9 and IRX10 genes in endosperm [8]. Consistent with this, the suppression of either GT43_2 or GT47_2 by RNAi in wheat endosperm led to up to 50% decrease in total AX content [10] while a triple mutant knockout of the three homeologues of GT43_2 (or TaIRX9b) had a 35% decrease in total AX [15]. Although the role of GT43_1 in wheat endosperm has not so far been confirmed the GT43_1 protein has been identified as being a key component of a xylan synthase complex from wheat seedlings ([13]; called “TaGT43-4” in their nomenclature) as, unlike GT43_2 and GT47_2, it is highly expressed in vegetative tissues. It is also not known whether the three subunits show any subtle differences in their roles; for example there are other homologues of IRX9, IRX14 and IRX10 expressed in wheat endosperm (albeit at lower levels than GT43_1, GT43_2, GT47_2) [8] and it is possible that the GT43_1, GT43_2, GT47_2 proteins could participate in different xylan synthase complexes which synthesise different forms of AX.

In order to explore the role of different forms of xylan synthase in determining xylan structure we have therefore generated two series of transgenic lines, with down-regulation of either GT43_1 or of all three xylan synthase genes (GT43_1+ GT43_2+ GT47_2), and determined the effect on xylan structure and other aspects of grain development and composition. This knowledge will complement our previous work on GT43_2 and GT47_2 and complete our understanding of highly expressed genes controlling xylan backbone synthesis in wheat starchy endosperm cell walls.

## 2 Materials and methods

### 2.1 RNAi constructs and transformation of wheat lines

An RNAi construct to down-regulate the three homeologues of GT43_1, under the control of the starchy endosperm-specific *HMW1Dx5* promoter was created using a *Bgl*II/*BamH*1 cloning strategy as described by [16], using 538bp fragments from the cDNA sequence of the GT43_1 (+1153-+1690). All three homeologues (nucleotide and amino acid sequences) i.e. TraesCS7A02G441400, TraesCS7B02G340100, and TraesCS7D02G430700 with the targeted sequence highlighted for nucleotide sequences are shown in S1 Fig. The fragment was generated by PCR (Phusion *Taq* polymerase from Thermo Scientific) using PCR primers GT43_1RNAiF and GT43_1RNAiR (S1 Table). An alignment of the nucleotide sequences of the three wheat homeologues for GT43_1 is shown in S2A Fig. Alignment of the targeted sequence for all three homeologues by the RNAi construct is shown in S2B Fig respectively. Generation of the GT43_2 and GT47_2 RNAi constructs have been previously described [10].

Sequencing of the constructs was carried out using the BigDye Terminator Version 3.1 Cycle Sequencing Kit (Applied Biosystems- Lingley House, 120 Birchwood Blvd, Cheshire, Birchwood, Warrington, WA3 7QH, UK), with construct specific primers; M13F, Rab1 and adh3R (S1 Table) used to confirm orientation of RNAi fragments. All reactions were analysed at Source Bioscience (1 Orchard Place, Nottingham Business Park, Nottingham NG8 6PX, UK).

For the generation of single GT43_1 RNAi knockdown lines and GT43_1+ GT43_2+ GT47_2 RNAi triple knockdown lines, wheat transformation was carried out by particle bombardment (PDS1000; Bio-Rad) of immature scutella. Scutella from immature caryopses of cv. Cadenza at ten to fourteen days post-anthesis (dpa) were co-bombarded with the pAHC20 plasmid, containing the selectable marker gene *bar* driven by the constitutive ubiquitin promoter from maize, as described by [17].

Genomic DNA was extracted from leaf material (Promega Wizard genomic DNA purification kit; Nuclei Lysis Solution A7943; Protein Preciptation Solution A7953; Promega UK Ltd- 2 Benham Road, Southampton Science Park, Chilworth Southampton, Hampshire, SO16 7QJ, UK) per the manufacturer’s instructions. Transgene presence was confirmed by PCR using appropriate primer combinations shown in S1 Table. The reactions were performed in a total volume of 25μL using a 1.1X ReddyMix™PCR Master Mix containing 1.5 mM MgCl_2_ (Thermo Scientific- Stafford House, 1 Boundary Park, Hemel Hempstead Industrial Estate, Hemel Hempstead HP2 7GE, UK) (~200ng of genomic DNA and 0.8μM of each primer). The cycling conditions were 96°C for 5 min followed by 32 cycles of 96°C for 30 s; 58°C for 30 s; 72°C for 1 min 30 s and the extension of 72°C for 10 min for PCR reactions. PCR products were analysed on 1.0% (w/v) agarose gels, stained with ethidium bromide and visualised by UV light.

### 2.2 Plant growth

Homozygous and azygous (null) T3 segregants for the single GT43_1 RNAi transgenics descended from the same original RNAi transformants and triple transgenic lines (T3) with controls were grown in four replicate pots, with four plants per pot, in a four block design (one replicate of each line i.e. transgenic and null per block) in temperature controlled GM glasshouse rooms with 18°C to 20°C day and 14°C to 16°C night temperatures and a 16-h photoperiod provided by natural light supplemented with banks of Son-T 400 W sodium lamps (Osram, Ltd) giving 400 to 1,000 μmol m^-2^ s^-1^ photosynthetically active radiation.

### 2.3 Zygosity determination of plant lines

Quantitative real time PCR analysis using TaqMan chemistry was used to estimate the numbers of transgene copies in individual plants, similar to [18]. An amplicon from the GT43_2, GT47_2 and/or GT43_1 (with a FAM reporter) and an amplicon from a wheat internal positive control (IPC, with a VIC reporter) were amplified together in a multiplex reaction (15 minutes denaturation followed by 40 cycles of 15 seconds at 95°C and 60 seconds at 60°C in an Applied Biosystems ABI7900 or QuantStudio 5 realtime PCR machine. Two replicate assays were run per sample. Fluorescence from the FAM and VIC fluorochromes was measured during each 60°C step, and the Ct values obtained. The difference between the Ct values for the GT43_2, GT47_2 and/or GT43_1 and the IPC (the DeltaCt) was used to classify the samples into groups with the same gene copy numbers. All RNAi lines were analysed with the QuantStudio (Thermo Fisher Scientific, Stafford House, 1 Boundary Park, Hemel Hempstead Industrial Estate, Hemel Hempstead HP2 7GE UK). Zygosity testing of all lines was carried out at IDna Genetics Ltd (The Norwich Bioincubator, Norwich Research Park, Norwich, NR4 7UH, UK).

### 2.4 RNA extraction and quantitative Real Time-PCR analyses of transgenic wheat and control lines

Tissue samples enriched in starchy endosperm cells were isolated from developing grain of T3 wheat lines at 15 dpa by gentle squeezing to release the endosperm tissue from the pericarp. RNA was extracted as reported by [19] and DNase (Promega UK Ltd., 2 Benham Road, Southampton Science Park, Chilworth Southampton, Hampshire, SO16 7QJ, UK) treated as per manufacturer’s instructions, to remove contaminating DNA. First-strand cDNA synthesis was carried out with a Superscript III Reverse Transcriptase Kit (Invitrogen- Invitrogen Ltd, Unit 3, Fountain Drive, Inchinnan Business Park, PA4 9RF Inchinnan, Renfrewshire, Scotland). The cDNA synthesis step was carried out as follows in a final 20μL volume: 2μg equivalent of DNase treated RNA; adjusted to a 12μL with sterile distilled water, 1μL of 100μM Oligo-dT primer, 1μL of 10mm dNTPs; 65°C for 5 mins followed by 1 min incubation on ice. This was followed by the addition of 4μL of 5x first strand cDNA synthesis buffer, 1μL of 0.1M DTT and 1μL of Superscript III Reverse Transcriptase; 50°C for 60 mins followed by 70°C for 15 mins.

Transcript levels were measured using the SYBR Green Jumpstart™ Taq Readymix™ for quantitative Real Time-PCR (qRT-PCR; Sigma- Merck Life Science UK Limited, The Old Brickyard, New Rd, Gillingham, Dorset, SP8 4XT, UK). The reaction was carried out in a 20μL mixture containing 1xGreen Jumpstart™ Taq Readymix™, 5μL 0.4μM of each primer with the ROX reference dye provided, 5μL cDNA working volume (1:15 dilution of original cDNA reactions). The following temperature profile was used: an initial denaturation step of 95°C for 10 min, followed by 40 cycles of 95°C for 15 s and 60°C for 1 min with an added dissociation stage of 95°C 15 s; 60°C for 1 min; 95°C for 15 s and 60°C for 15 s. All reactions were carried out in 96 well plates and analysed using the Applied Biosystems 7500 real-time PCR instrument and software v2.0.5. Transcript levels for GT43_1 RNAi and triple knockdown RNAi lines were determined using the primers shown in S1 Table. Three reference genes were used to normalize expression: *Ta*2526 (Prosm), a stably expressed EST from grain (primers prTYW19 and TYW20), glyceraldehyde-3-phosphate dehydrogenase (primers prTYW422 and prTYW423) (*Ta*GAPDH), and succinate dehydrogenase (primers prTYW424 and prTYW425) (*Ta*SDH). All primer sequences are given in S1 Table.

Following qRT-PCR, normalised relative quantity (NRQ) data were calculated for the target genes of interest by using the formula: *NRQ* = *E*_*T*_^-*ctT*^/ (*E*_*R1*_^-*ctR1*^. *E*_*R2*_^-*ctR2*^.*E*_*R3*_^-*ctR3*^) [20]; where *E*_*T*_, *E*_*R1*_, *E*_*R2*_, *E*_*R3*_ are the efficiencies of the target (GT43_1, GT43_2 or GT47_2) and the three reference genes used (Prosm, *Ta*SDH and *Ta*GAPDH); *ctT*, *ctR1*, *ctR2*, *ctR3* are the corresponding numbers of cycles at threshold fluorescence set for the reactions; and the denominator of the expression is the geometric average of the relative expression of the three reference genes. As these data have heterogeneity of variance, a transformation, log_2_(1/NRQ) [21], was applied prior to analysis. This transformation provides data on the ct-scale.

### 2.5 Preparation of flour for biochemical analyses

Ten grams of seed from each transgenic and control line were analysed using a NIRFlex solids BÜCHI machine and the NIRWare 1.2 Software (BÜCHI UK Ltd, Unit 6 Goodwin Business Park, Willie Snaith Road, Newmarket, CB8 7SQ, UK), using an internal calibration for seed moisture content. The moisture content was adjusted to 15.5% by the addition of water and samples were conditioned for 2 hours at room temperature on a Rolling Shaker before being milled using a Micro Scale Labmill FQC-2000 (METEFÉM SZÖVETKEZET, Hungary, 1047 Budapest, TINÓDI U. 28–30.). This small-scale roller mill has a similar action to large commercial roller mills. Wholegrain flour from the mill was sieved into three fractions corresponding to bran, flour of ≤ 250μM and white flour of ≤ 150μM. All biochemical analyses were carried out on the ≤ 150μM fraction.

### 2.6 Enzyme fingerprinting of arabinoxylan and β-glucan

Arabinoxylan and β-glucan structures were determined by enzyme fingerprinting [22]. Briefly, 100 mg of wheat flour was resuspended in 1 mL of 80% ethanol (v/v) and heated at 95°C for 10 min to inactivate endogenous glycosyl hydrolyses. Samples were centrifuged for 2 min at 13,400x*g*. Supernatant was discarded and the washing step was repeated with 80% and finally 95% ethanol wash. The pellet was dried for 30 min *in vacuo*. Two μL of endoxylanase (NpXyn11) and 1 μL of lichenase (CtGH26; both Prozomix) were added and made up to 1 mL with water. The overnight incubation was carried out at 40°C in Thermomixer (Eppendorf) under constant shaking at 750 rpm. Samples were centrifuged (13,400x*g* for 5 min), the supernatant collected and boiled for 30 min to inactivate the fingerprinting enzymes. Samples were diluted 1:20 in 10μM melibiose (as an internal standard) in water and filtered using 0.45 μm PVDF filters (Whatman) prior injection onto HPAEC-PAD (Dionex ICS-3000; Thermo Scientific) system equipped with CarboPac PA-1 guard (2x50 mm) and analytical (2x250 mm) column. The fingerprinting generated AX and β-glucan oligosaccharides were separated using method developed by [23] and modified by [16]. Chromeleon (v. 7.2SR4; Thermo Scientific) was used to interpret the data. The amount of each AX oligosaccharide (for used nomenclature see [24]) and G3 and G4 oligosaccharides derived from β-glucan were calculated as measures of enzyme-extractable AX and β-glucan, respectively.

### 2.7 Hydrolysis of wheat flour and quantification of neutral monosaccharides by HPAEC-PAD

Fifty μg of wheat flour was hydrolysed in 400 μL of 2M triflouroacetic acid (TFA; Sigma) for 1 h at 120°C. Hydrolysed samples were cooled, dried *in vacuo*, washed in 500 μL water and dried again. Finally, the pellet was redissolved in 500 μL of water and samples were stored in -20°C until analysis. Calibration curves (25–625 pmol) were generated for each monosaccharide prepared following TFA treatment as described. Neutral monosaccharides were separated by HPAEC-PAD using Dionex ICS-5000+ equipped with KOH eluent generator and CarboPac PA-20 column (guard 3x30 mm; analytical 3x150 mm; Thermo Scientific) using method developed in [25]. Chromeleon software was used to mark and quantify the peaks.

### 2.8 Measurements of seed parameters

The areas, lengths and widths of mature seeds were measured using the MARVIN- Digital Seed Analyser (MARViTECH GmbHGermany) and software Marvin 4.0. Grain hardness, individual seed weight and diameter were determined using the Perten Single Kernel Characterisation System (SKCS) 4100 (Calibre Control International Ltd, 5–6 Asher Court, Lyncastle Way, Warrington, WA4 4ST, UK) following the manufacturer’s procedure. Three hundred grains for each plant from each block were used for each analysis (Perten Instruments, Calibre Control International Ltd, UK).

### 2.9 Statistical methodology

Statistical analyses were performed using Genstat 20^th^ Edition (VSNi). Some response variables required square root or log_e_ transformations to satisfy the normality and homogeneity of variance assumptions. The transformations used for each variable are indicated in the results section/tables.

### 2.10 Fingerprinting, NMR and monosaccharide data

The fingerprinting, NMR and monosaccharide data were analysed with ANOVA. GT43_1 RNAi single line data (Lines 1–6) was firstly analysed with treatment structure type*line where type is included to compare the average of all transgenic lines to the average of all nulls, line is included to compare the averages of each line (across transgenic and null) and the interaction (type.line) tests whether the difference between transgenic and null is consistent across the 6 lines. These data were also analysed with 6 pairwise contrasts comparing each line to its corresponding null directly (NB the replication in this experiment is within plant and is therefore pseudo-replication. Any conclusions drawn apply only to the plants included in the experiment and not the population of all possible plants).

The Triple line (Lines 7 and 8) data were analysed with PlantID included as a block effect and observations within plant were considered to be pseudo replication (so there were 3 true reps per treatment and 3 pseudo reps within each rep). The treatment structure used was Type/LineNo where Type compares the average of the two triples to the average of the controls and Type.LineNo compares the average of line 7 to the average of line 8.

A further ANOVA style analysis was used to compare the single GT43_1 RNAi transgenic lines to Triples and the effects of transgenic lines to the effect of nulls. This was performed using the REML directive in order to get predictions from the unbalanced structure. PlantID was included as a block effect (random structure) and observations within plant were considered to be pseudo replication (so there were 6 true reps of Single-transgenic, single-null and triple-transgenic but only 3 true reps of triple-null). The treatment structure (fixed structure) was (Type1*Type2)/Number which gives the following four tests. Type1 tests for a difference between singles and triples (averaged across all lines and nulls), Type2 tests for a difference between transgenics and nulls (averaged across singles and triples) and the interaction testes whether the difference between transgenic and null is different for singles and triples? The term Number is included for completeness of the structure to make sure comparisons are made against correct background variation by accounting for any differences between lines within each combination.

### 2.11 qPCR data

The singles data were log_10_ transformed and analysed using ANOVA with treatment structure Line*Type where Line tests for any differences between the 6 lines (averaged across null and transgenic), Type tests whether there is any evidence that nulls and transgenics differ (averaged across the 6 lines) and the interaction tests whether the differences between null and transgenic differ for different lines?

For the triples data, GT43_1, GT43_2 and GT47_2 were all square root transformed and then analysed using ANOVA with block structure IDNo/SampleType taking account of different levels of replication and pseudo-replication. The treatment structure was Line*Type giving the same set of tests as for the singles.

### 2.12 Seed parameters

The seed parameter data were all analysed using ANOVA. The treatment structure for the singles data was Line*Type where Line tests for any differences between the 6 lines (each averaged over T and N), Type tests for a difference between N and T (averaged over the 6 lines) and the interaction tests whether the differences between N and T are different for the different lines?

The treatment structure for the triples was Type/(lineT + lineC) where Type tests for a difference between control and transgenic, lineT tests for a difference between line 7 and 8 and lineC tests for any differences between the 3 controls.

### 2.13 Microscopy

Wheat grains were fixed in 4% (w/v) paraformaldehyde, 2.5% (w/v) glutaraldehyde and dehydrated in a graded ethanol series. The samples were then processed through increasing concentrations of LR White resin (Agar Scientific UK, R1281) and embedded at 58°C for 16–20 h in a nitrogen-rich environment [26].

One μm sections of the resin blocks were cut with a Reichert-Jung ultramicrotome and dried onto Polysine coated slides (Agar Scientific UK, L4345) at 40°C. Sections were immunolabelled as described by [26] using the primary antibody LM11 (from rat) and Alexa Fluor 488 goat anti-rabbit IgG (Invitrogen A11008).

The LM11 monoclonal antibody (kindly provided by Professor Paul Knox, University of Leeds) was generated using a neoglycoprotein (xylopentaose-BSA) and is a high affinity antibody to the non-reducing end of (1,4)-β-D-xylosyl residues that constitute the backbone of xylans. LM11 antibody can bind strongly to xylans that have a higher degree of substitution of the xylan backbone such as wheat arabinoxylan where the xylan backbone is substituted with sidechains of arabinofuranosyl residues. Images were acquired with a Zeiss LSM 780 confocal microscope using Zeiss ZEN 2010 software.

## 3 Results

### 3.1 Generation and characteristics of transgenic lines

Six independent transgenic lines (T1—T6) were regenerated from immature embryos after bombardment with a RNAi construct for GT43_1, with corresponding null (azygous) control lines (N1-N6) selected by segregation of the progeny of the heterozygous T1 plants (Table 1). To generate triple transformants expressing RNAi constructs for the GT43_1, GT43_2 and GT47_2 genes, all three RNAi constructs were co-bombarded (in a 1:1:1 molar ratio). Two triple transformed lines were generated (T7 and T8) and three T1 sister lines from each of these (T7-1 to T7-3; T8-1 to T8-3). Three control lines (C1-3) were also regenerated from immature embryos subjected to the same bombardment conditions as the T7 and T8 lines (Table 1).

**Table 1:** Characteristics of GT43_1 single and GT43_1+GT43_2+GT47_2 Triple RNAi lines and control lines, including Plant code, Transgenic code, RNAi construct, Zygosity and differences compared to control data.

Plant code	Transgenic code	RNAi construct	Copy number of homozygotes	Zygosity	Percentage of transcript compared to control (100%)
Line 1 T	B3120 R9P5	GT43_1	4	H	41.22±8.45
Line 2 T	B3213 R5P5	GT43_1	2	H	34.53±4.87
Line 3 T	B3264 R1P8	GT43_1	22	H	13.93±2.30
Line 4 T	B3264 R6P1	GT43_1	6	H	37.11±6.67
Line 5 T	B3289 R1P2	GT43_1	6	H	33.42±7.33
Line 6 T	B3289 R3P1	GT43_1	18	H	31.29±3.27
Line 7 T	B3395 R10P4.6	GT43_1	4	H	58.46±10.55
	(12-T2 plants tested)	GT43_2	1–2	S	53.79±8.43
		GT47_2	8–16	S	117.07±31.19
	B3395 R10P4.14	GT43_1	4	H	44.08±5.72
	(12-T2 plants tested)	GT43_2	2	H	34.34±13.37
		GT47_2	16	H	37.19±4.70
	B3395 R10P4.18	GT43_1	4	H	65.40±7.81
	(12-T2 plants tested)	GT43_2	2	H	12.35±2.29
		GT47_2	16	H	17.98±2.25
Line 8 T	B3395 R11P3.2	GT43_1	12	H	41.21±4.08
	(12-T2 plants tested)	GT43_2	3	S	21.54±5.98
		GT47_2	16	H	23.90±2.52
	B3395 R11P3.13	GT43_1	12	H	59.52±2.98
	(12-T2 plants tested)	GT43_2	3	S	62.62±6.48
		GT47_2	16	H	20.48±8.23
	B3395 R11P3.15	GT43_1	12	H	16.52±3.00
	(12-T2 plants tested)	GT43_2	3	S	26.68±5.59
		GT47_2	16	H	17.21±3.04

For the Plant code, transgenic codes and Zygosity numbering system used is explained as follows: B = Bombardment Number; R = Replicate Number; P = Plant Number; H = Homozygous; S = Segregating; T = Transgenic.

The abundances of GT43_1 transcripts in developing endosperms (15 dpa) of the transgenic and null segregants from the same transformation events were determined by quantitative reverse transcription-PCR. Successful transformation events were detected by differences in transcript levels from the corresponding null controls (Table 1 and S3 Fig).

Lines T1-6 were homozygous for the GT43_1 transgenes, with copy numbers ranging from 4 to 22 and transcript levels from 14% to 41% of those in the corresponding null lines (Table 1). ANOVA was used to compare the mean values for T1-T6 to those for N1-N6 (called “type”). In addition, the six pairs of lines (T1 and N1, T2 and N2 etc) were compared to determine whether differences occurred which were related to the transformation and regeneration of the plants (called “line”). Finally, the interaction between type and lines was tested to determine whether the differences between types (T and N) were consistent between all pairs of lines (called type.line) (S2 Table). This showed a significant difference between the nulls and transgenic lines (p<0.001) with transgenic lines having lower values of transcripts. There were also significant differences in transcript levels between the 6 transgenic lines (p<0.001); line 2 had significantly higher transcript levels than the others while line 6 had significantly lower values (S3 Fig). There were no significant interactions between transgene and line effects (p = 0.66) suggesting that the knockdown of transcript levels due to RNAi construct, between transgenic and null is similar for all lines. L3 line had both the greatest reduction in transcript levels and the highest transgene copy number (22).

Lines T7-2 and T7-3 were homozygous for all three transgenes (GT43_1, GT43_2 and GT47_2 RNAi) while the other T7 and T8 lines segregated (i.e. were heterozygous) for at least one transgene. Transgene copy numbers ranged from 2 to 16. Transcript levels varied widely, from little or no impact of the transgene (GT47_2 in T7-1) to less than 20% of the levels in C1-3 (GT47_2 in T7-3 and T8-3). Transcript levels were similar for all 3 target genes (i.e. GT43_1, GT43_2 and GT47_2) (S3 Table). The nulls and transgenics are significantly different with the controls having higher values than transgenics (p = 0.042, 0.015, 0.007 respectively) (S3 Table). There was no significant interaction between line and transgene effects on any of the target genes (S3 Table) suggesting that the magnitude of the knockdown of transcript levels due to all three RNAi constructs are similar for lines 7 and 8. The decreases observed for the three types of transcripts were by 1.6–2.3 fold for GT43_1, 1.9–8.3 fold for GT43_2- and 2.8–5.9 fold for GT47_2 for line 7, and by 1.7–7.3 fold for GT43_1, 1.7–6.3 fold for GT43_2 and 4.3–6.4 fold for GT47_2 for line 8 although these differences between lines were not detected as significant.

### 3.2 Effects of RNAi down-regulation on grain composition

All constructs were expressed under the control of the HMW subunit 1Dx5 promoter, which is only expressed in the starchy endosperm cells of the developing grain [27]. This tissue forms the white flour fraction on milling so the lines were milled using a small scale roller mill and white flour fractions analysed. GT43_1, GT43_2 and GT47_2 are likely to encode subunits of xylan synthase which form a trimeric complex *in planta* [11, 12]. Analyses of the lines therefore focused on the amount and composition of arabinoxylan using three approaches. Firstly, enzymic fingerprinting was used to determine the amount and structure of AX and β-glucan (mixed linkage glucan, MLG), the first and second most abundant cell wall polysaccharides in white flour, respectively. Secondly, a fraction comprising cell wall polysaccharides was extracted and the monosaccharide composition determined following mild acid hydrolysis. Because xylose is only present in significant amounts in wheat flour as AX, the contents of total and soluble xylose (X) were used as measures of AX (arabinose was not used for this measure because the contribution of arabinogalactan peptide (AGP) to total arabinose was not quantified). Thirdly, soluble sugars (glucose, sucrose, maltose and raffinose) were determined by ^1^H NMR spectroscopy after extraction with deuterated methanol:water.

### 3.3 Effects on arabinoxylan and β-glucan polysaccharides

The contents of enzyme-extractable AX and mixed-linkage glucan (MLG) determined by fingerprinting and of total and soluble X determined by monosaccharide analysis in the transgenic and null lines are summarised in Table 2 and presented in full in S4 Table. This shows similar effects in both the single (GT43_1) and triple (GT43_1, GT43_2, GT47_2) transgenics. The amounts of xylanase-extractable AX determined by fingerprinting are decreased to 33% and 35% of the control values and of total X determined by monosaccharide analysis to 46% and 47% of the control values, respectively. The effect on soluble X was greater in both sets of lines, which was decreased to 20% and 21% of the control values, than on total X. This resulted in increased ratios of total to soluble X, from 6.5 to 17.7 in lines T1-6 and from 4.7 to 15.4 in the triple lines T7 and T8. Increases in the amounts of MLG occurred in both RNAi lines (both single and triple lines), by 148% in T1-6 and 215% in T7 and T8, resulting in decreased ratios of AX:MLG (7.44 to 1.74 and 5.89 to 1.96, respectively). ANOVA was used to compare the compositions of the transgenic and null lines as described above for the qRT-PCR analysis (Table 3, S5 Table). This showed significant differences between contents of AX and MLG in the T and N lines which were consistent between pairs. However, differences between the 6 pairs were also observed, demonstrating the importance of comparing transgenic lines with null controls.

**Table 2:** Amounts of arabinoxylan and β-glucan (MLG) detected in the GT43_1 single and Triple RNAi lines compared to their respective null and control lines.

	GT 43_1 lines		Triple Lines	
	T1-T6	N1-N6	T7-T8	C1-3
	mean ± SD	mean ± SD	mean ± SD	mean ± SD
TOT-AX (AU)* % control	4.01±0.760 33.0	12.16±0.772	3.49±0.871 34.9	9.99±2.705
TOT-X (mg/g)** % control	5.38±1.279 45.9	11.71±1.227	8.32±1.114 47.4	17.55±0.930
WE-X (mg/g)** % control	0.38±0.222 20.6	1.84±0.218	0.79±0.483 19.9	3.97±1.006
Ratio TOT-X:WE-X** % control	17.69±7.888 273.4	6.47±1.173	15.41±9.393 330.7	4.66±1.144
Ratio WE-X:TOT-X** % control	0.07±0.038 43.8	0.16±0.030	0.10±0.065 45.5	0.22±0.050
TOT-MLG (AU)* % control	2.49±0.613 148.2	1.68±0.323	3.61±0.424 214.9	1.68±0.317
Ratio AX:TOT-MLG*	1.74±0.708 23.4	7.44±1.327	0.96±0.192 16.3	5.89±0.875

*Enzyme extractable fractions from fingerprinting

**From monosaccharides

**Table 3:** Probabilities (p) from ANOVA of differences in amounts, ratios, and compositions of AX and MLG in transgenic and control lines. Transformed data (log or sq. root) were used for some data as detailed in S4 Table.

	GT-43_1 transgenics (T1-6) and null controls (N1-6)			Triple transgenics (T7+ T8) and controls (C1-3)		GT-43_1 vs Triple transgenics
	Type AvN1-6 v AvT1-6	Lines Are there differences between means of pairs of lines (T1/N2, T2/N2 etc).	Type.Line Are the differences between T/N lines consistent	C1-3 v T7+T8	T7 v T8	Are the differences between T1-6 and N1-6 different to those between T7+8 and C1-3.
Amounts and ratios of AX and MLG						
TOT-AX	<0.001	<0.001	0.105	<0.001	0.217	0.476
TOT-X	<0.001	0.002	0.053	<0.001	0.787	0.054
WE-X	<0.001	<0.001	<0.001	0.002	0.894	0.308
WE-X:TOT-X	<0.001	<0.001	0.002	0.040	0.824	0.620
TOT-MLG	<0.001	<0.001	0.001	<0.001	0.246	0.296
TOT-AX:TOT-MLG	<0.001	<0.001	<0.001	<0.001	0.379	0.063
Structure of AX						
Xylose	<0.001	<0.001	0.016	0.004	0.54	0.801
Xyl2	<0.001	<0.001	0.047	<0.001	0.378	0.015

ANOVA of the averages of the two triple transgenic lines (T7, T8) and the averages of these lines to the average of the controls (C1-3) (Table 3 and S6 Table) showed no significant differences between T7 and T8, but that these lines differed significantly from C1-3 in the amounts of AX, MLG as well as the ratio WE-X:TOT-X (P = 0.040).

**Fig 1:**
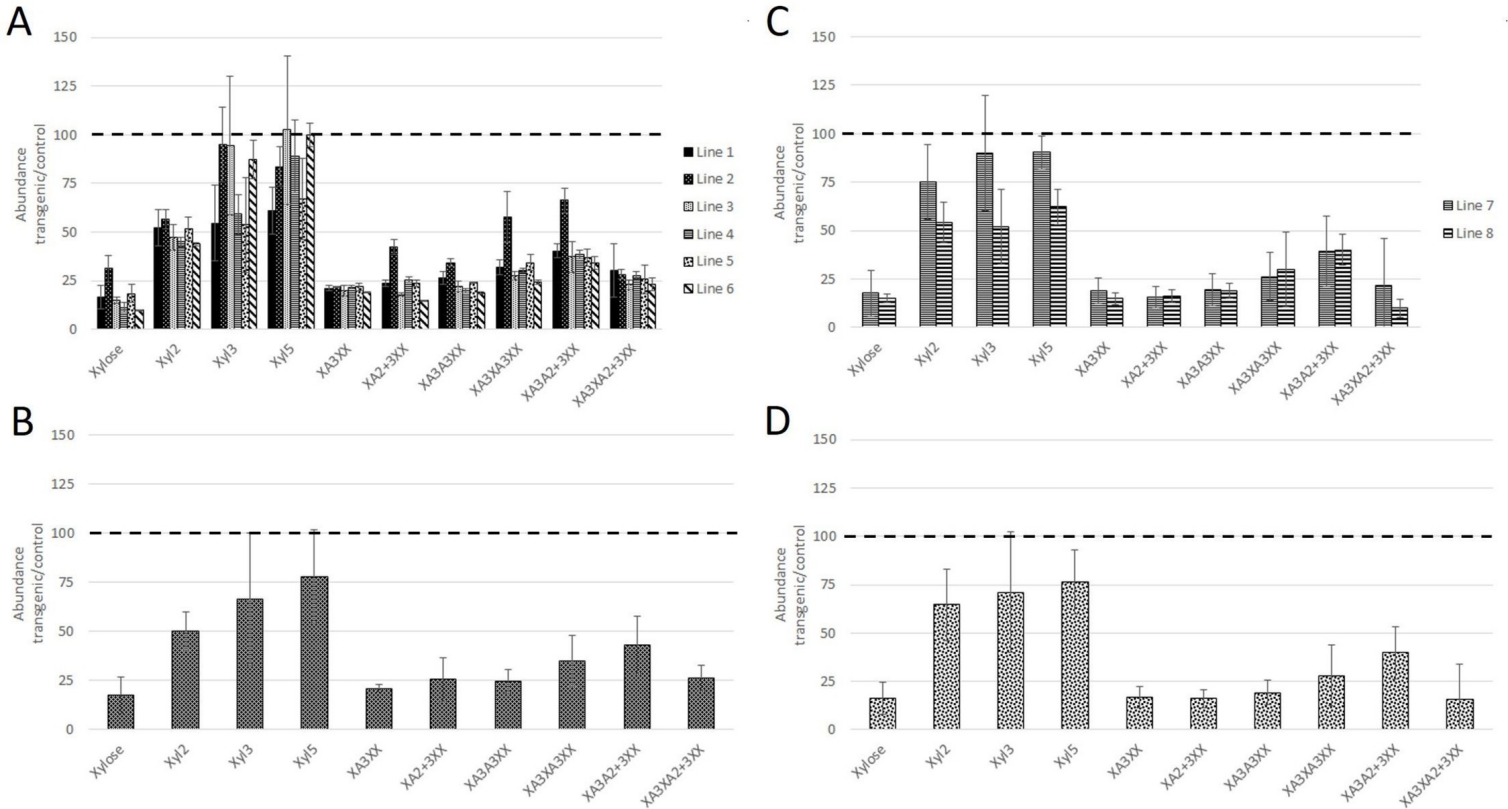
Relative abundances (transgenics compared to controls) of oligosaccharides (AXOS) released by digestion of arabinoxylan (AX) in wheat flour with endoxylanase and separation by High Performance Anion Exchange Chromatography (HPAEC). A, lines T1-6 (GT43_1 RNAi) compared with respective null controls N1-6; B lines T7 and T8 (GT43_1+GT43_2+GT47_2- triple RNAi) compared individually with the means of lines C1-3; C, means of lines T1-6 compared with means of lines N1-6: D, means of lines T7 and T8 compared with means of lines C1-3.

Analysis of AX structure by fingerprinting showed differences between the T and N lines, with greater decreases in the amounts of substituted AXOS compared to xylan fragments derived only from the xylan backbone (Xyl 1, 2, 3 and 5) (S7 Table). These differences were statistically significant between types of lines (T and N) for all AXOS except XA3XA3XX and there were significant differences between the six between pairs of lines for all AXOS except XA3XA2+3XX (Table 3 and S8 Table). These effects are illustrated in Fig 1, with the peak areas of AXOS released by digestion with endoxylanase of the T lines being expressed as percentages of those in the corresponding N lines and panels A and C showing the individual lines and means, respectively.

Analysis of the triple transgenic lines showed similar effects with greater reductions in substituted AXOS than those derived solely from xylan backbone (Xyl, Xyl2, Xyl3, Xyl5) (Fig 1 Panels B and D). Similarly, ANOVA showed that the amounts of AXOS differed significantly between the transgenic and control lines, but not between the T7 and T8 lines (except for the AXOS, XA3XA3XX and XA3A2 + 3XX) (Table 3, S9 Table).

The data above therefore indicate that the GT43_1 and triple transformations had similar effects on cell wall polysaccharides. ANOVA was therefore used to compare the two types of transgenic line, asking whether the two sets of lines showed significant differences when compared with their control lines (ie. T1-6 v N1-6, T7/8 v C1-3) (Table 3, S10 and S11 Tables). No significant differences in effects on the amounts of cell wall polysaccharides or amounts of AXOS were observed, indicating the suppression of the single GT43_1 gene and of the three xylan synthase genes together (GT43_1, GT43_2, GT47_3) had similar effects.

### 3.4 Effects on sugars

NMR spectroscopy was used to determine the amounts of the major soluble carbohydrates in the flour samples: the monosaccharide glucose, the disaccharides sucrose and maltose and the trisaccharide raffinose (Table 4A), and the lines compared by ANOVA (Table 4B, S12 Table). This showed increases in the concentrations of all sugars in the triple T7/8 lines relative to the control lines. By contrast, both sucrose and glucose were significantly lower in the GT43_1 lines T1-6 than in N1-6 (representing 53% and 79% of the control concentrations). The effect of GT43_1 RNAi and triple RNAi transgenes on these sugars were therefore quite different, unlike the results for AX (Table 5).

**Table 4:** Determination of soluble sugars by NMR spectroscopy for GT43_1 single and Triple RNAi lines compared to their Null and Controls respectively.

**A.** Amounts determined as				
	GT 43_1 lines		Triple Lines	
	T1-T6	N1-N6	T7-T8	C1-3
	Mean ± SD	Mean ± SD	Mean ± SD	Mean ± SD
Raffinose (mg/g)% control	0.80 ± 0.145 96	0.83 ± 0.315	1.12 ± 0.289 184	0.61 ± 0.069
Maltose (mg/g) % control	23.96 ± 12.934 104	23.08 ± 17.668	43.22 ± 13.354 327	13.20 ± 7.389
Sucrose (mg/g) % control	5.22 ± 1.480 53	9.92 ± 3.824	10.34 ± 4.080 209	4.94 ± 0.744
Glucose (mg/g) % control	1.72 ± 1.312 79	2.18 ± 0.665	3.14 ± 1.019 378	± 0.119

**B.** p values from ANOVA of sugars determined by NMR.						
	GT-43_1 transgenics (T1-6) and null controls (N1-6)			Triple transgenics (T7+ T8) and controls (C1-3)		GT-43_1 vs Triple transgenics
	Type AvN1-6 v AvT1-6	Lines Are there differences between means of pairs of lines (T1/N2, T2/N2 etc).	Type.Line Are the differences between T/N lines consistent	C1-3 v T7+T8	T7 v T8	Are the differences between T1-6 and N1-6 different to those between T7+8 and C1-3.
Raffinose	0.752	0.727	0.191	0.001	0.064	0.005
Maltose	0.699	0.085	0.019	0.003	0.244	0.013
Sucrose	<0.001	<0.001	<0.001	0.007	0.013	<0.001
Glucose	<0.001	0.007	<0.001	<0.001	0.131	<0.001

**Table 5:** ANOVA of sugars determined by NMR for GT43_1 and Triple RNAi lines compared to their Null and Controls respectively.

	GT-43_1 transgenics (T1-6) and null controls (N1-6)			Triple transgenics (T7+ T8) and controls (C1-3)		GT-43_1 vs Triple transgenics
	Type AvN1-6 v AvT1-6	Lines Are there differences between means of pairs of lines (T1/N1, T2/N2 etc)?	Type.Line Are the differences between T/N lines consistent	C1-3 v T7+T8	T7 v T8	Are the differences between T1-6 and N1-6 different to those between T7+8 and C1-3?
raffinose	0.752	0.727	0.191	0.001	0.064	0.005
Maltose	0.699	0.085	0.019	0.003	0.244	0.013
Sucrose	<0.001	<0.001	<0.001	0.007	0.013	<0.001
Glucose	<0.001	0.007	<0.001	<0.001	0.131	<0.001

### 3.5. Effects on seed parameters

Image analysis was used to measure grain size and shape (grain area (mm^2^), length (mm) and width (mm)) and the Perten SKCS to determine grain weight, hardness, diameter and moisture content. Full details of these are provided in (S13–S17 Tables).

No significant differences were observed for the area, length, width, diameter and weights of the GT43_1 transgenic grains (T1-6) compared to their null controls (N1-6) (S13 and S14 Tables; S5 Fig). However, the T1-6 grains were significantly softer than N1-6, by 6–11 units determined by the SKCS (p< 0.001). Grain moisture content was also significantly different between transgenic and null controls for GT43_1 lines (p = 0.004) (S15 and S16 Tables).

Significant differences were also observed between the triple transgenic lines (T7, T8) and the controls (C1-3) for moisture (p = 0.04), diameter (p = 0.013) and weight (p = 0.02), with the transgenic lines being smaller, and for grain hardness, with the transgenics being softer (p = 0.02) measured by the SKCS. Significant effects were also observed for seed area (p = 0.042) and seed width (p = 0.029) but not seed length (p = 0.067) between line 7 and 8 compared to the control lines, but no significant differences were observed between lines 7 and 8 (S16 Table). The triple transgenic lines also showed a wrinkled and collapsed seed phenotype compared to the control (S6 Fig).

There were also significant differences for all seed parameters between the GT43_1 transgenics and the triple transgenic lines, with the single transgenic lines showing greater reductions in all seed parameters (i.e. area p = 0.001; length p< 0.001; width p = 0.026; weight p< 0.001 and diameter p< 0.001) than the triple transgenic lines except for moisture (moisture p = 0.006) where the GT43_1 transgenics showed higher mature grain moisture contents than the triple lines. There was also a significance difference in grain hardness between the GT43_1 transgenics and the triple lines, with the latter being significantly softer than the single GT43_1 lines (p<0.001) (S17 Table).

### 3.6. Immunolocalisation of AX in starchy endosperm cell walls

**Fig 2:**
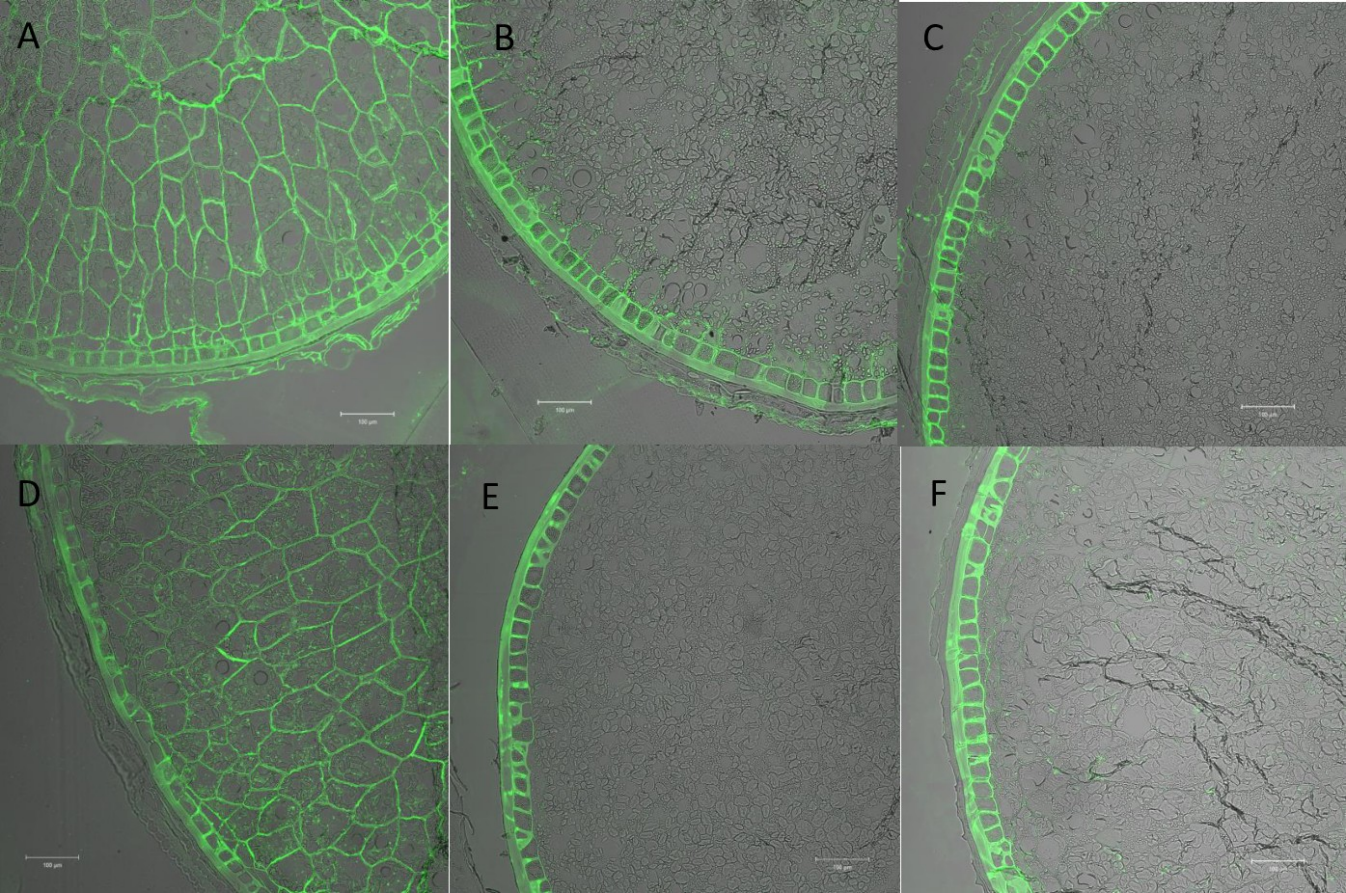
Immunolabelling of medial transverse sections of developing grain at 15 DAA of control and transgenic lines using the monoclonal antibody LM11 to show the amount and distribution of arabinoxylan. A-C, comparison of the null line N1 (A) and the GT43_1 RNAi lines T1 (B) and T5 (C); D-F, comparison of the control line C1 with the triple transgenic lines (GT43_1+GT43_2+GT47_2 RNAi constructs) T7 (E) and T8 (F). Bar—100μm.

Immunolabelling of sections of developing wheat caryopses with the LM11 monoclonal antibody clearly labelled the endosperm cell walls (Fig 2; panels A-F). Analysis of two GT43_1 RNAi lines (T1 and T5) (panels B and C) and two triple transgenic lines (T7, T8) (panels E, F) showed reduced labelling of the cell walls in the starchy endosperm, but not the aleurone layer, compared to the respective control lines (panels A and D), but no obvious differences in the intensity of labelling between the GT43_1 and triple lines. This is consistent with the tissue-specificity of the HMW subunit promoter used to drive the RNAi constructs and the similar levels of suppression of TOT-AX in the two sets of lines (Table 2).

## 4 Discussion

RNAi down-regulation of the single xylan synthase subunit encoded by GT43_1 and simultaneous down-regulation of all three subunits (encoded by GT43_1, GT43_2, GT47_2) resulted in similar decreases in total AX, to 46% and 47% of the total X determined by monosaccharide analysis (Table 2). This compares with previously reported RNAi down-regulation of single xylan synthase subunit genes (GT43_2 or GT47_2) which resulted in decreases in total AX [10] to 50–60% of those in the control lines and the development of a triple knock-out mutant of GT43_2 (stacking mutations in A,B,D homeologues of GT43_2A,B,D/TaIRX9b) in which total AX was reduced to about 65% of that in the control line [15]. The proportions of short AXOS released by GH11 digestion were decreased more in the single GT43_1 and triple lines (to 33%, 35%) lines than total AX, as was the proportion of WE-X (decreased to 21%, 20%) (Table 2). This was also the case in our previous studies of RNAi suppression of GT43_2 and GT47_2 and of a triple mutant of GT43_2, where suppression of these genes induced a change in AX composition such that the solubility and digestibility with the GH11 endoxylanase were decreased [10, 15, 28]. This could be because suppression of these genes knock-outs xylan synthase complexes which synthesis a specific type of AX, or because the loss of cell AX in the endosperm cell walls results in compensatory increases in AX cross-linking: there was evidence of such compensation in our previous studies [15, 29] but that does not rule out the first hypothesis.

The fact that similar effects were observed for the single and triple transgenic lines may suggest that a minimum level of AX, corresponding to about half of that in the control lines, is required for normal seed development, with greater reductions being lethal. Alternatively, there may be more than one xylan synthase complex present in endosperm cells; with one composed of the more highly expressed subunits which was targeted in these studies (GT43_2 (IRX9), GT43_1 (IRX14), GT47_2 (IRX10)) and others composed of subunits encoded by less highly expressed genes (IRX9 (GT43_6, GT43_3, GT43_4), IRX14 (GT43_10) and IRX10 (GT47_1, GT47_4)). These other xylan synthase complexes may be sufficiently abundant to maintain about half the normal synthesis of AX. The fact that there is no additional effect of including GT43_2 and GT47_2 RNAi over and above that of GT43_1 RNAi alone is consistent with this IRX14 subunit being the limiting factor in making functional xylan synthase complexes in plants with GT43_1 RNAi. This is consistent with the fact that there is less redundancy of IRX14 (only one other expressed gene, GT43_10) and the larger effect on AX amount seen here compared with our previous studies on GT43_2 and GT47_2 RNAi [10].

The triple transgenic lines showed quite different effects on soluble sugars (glucose, sucrose, maltose and raffinose), which were increased compared to the control lines, from the effects of GT43_1 RNAi in which the levels of sugars were unaffected (raffinose, maltose) or decreased (sucrose, glucose). Differences between effects of GT43_1 and triple RNAi were also seen for seed parameters, with the triple but not the GT43_1 lines having smaller seeds than the controls. Both sets of transgenic lines were softer than their respective controls, but this effect was greater for the triple transgenic lines than the single lines (by about 16 compare to 6–11 units measured by SKCS). This suggests that reduced levels of AX may affect the mechanical properties of the cell walls resulting in greater friability on milling.

Therefore despite the highly similar effects on most AX measurements, there is evidence of differences in the effects of GT43_1 and triple RNAi on grain parameters not directly related to the cell wall. We do not have an explanation for these but suggests the additional suppression of GT43_2 and GT47_2 may have an effect on pathways other than AX synthesis, or on AX properties not determined here which are having downstream effects on these other parameters.

## Supporting information

S1 Fig *Triticum aestivum* GT43_1 nucleotide sequences. (PDF)

S2 Fig a. *Triticum aestivum* GT43_1 nucleotide sequence alignment of the three homeologues. b. Alignment of nucleotide sequences from the three GT43_1 homeologues targeted by RNAi construct.
(TIF)

S3 Fig qPCR transcript level data for six independent GT43_1 RNAi wheat lines. (TIF)

S4 Fig qPCR transcript level data for two independent GT43_1+GT43_2+GT47_2 RNAi wheat lines. (TIF)

S5 Fig *Triticum aestivum* mature seeds from GT43_1 and Triple (GT43_1+GT43_2+GT47_2) RNAi transgenic lines compared to their controls. (TIF)

S6 Fig Collapsed seed structure from Triple (GT43_1+GT43_2+GT47_2) RNAi wheat lines compared to control. (TIF)

S1 Table Primer name and sequence Table. (TIF)

S2 Table Tables of p values (A), means and SEMs (B) from ANOVA analysis of GT43_1 transcript levels in N1-6 and T1-6 determined by qPCR analysis of RNA extracted from 15dpa developing grain.
(TIF)

S3 Table Tables of p values (A), means and SEMs (B) from ANOVA analysis of GT43_1, GT43_2 and GT47_2 transcript levels in two Triple RNAi wheat lines containing all three constructs determined by qPCR analysis of RNA extracted from 15dpa developing grain.
(TIF)

S4 Table Full data for Arabinoxylan content (AX) and Mixed Linkage Glucan (MLG) by High Performance Anion Exchange Chromatography fingerprinting and monosaccharide analysis. (TIF)

S5 Table Tables of p values (A), means and SEMs (B) from ANOVA analysis of Arabinoxylan (AX) and Mixed linkage Glucan (MLG) in N1-6 and T1-6.
(TIF)

S6 Table Tables p values, means and SEMs from ANOVA analysis of Arabinoxylan (AX) and Mixed linkage Glucan (MLG) in T7, T8 and C1-3. (TIF)

S7 Table Table of full Arabinoxylan Oligosaccharide (AXOS) and Mixed Linkage Glucan (MLG) data. (TIF)

S8 Table Tables of p values, means and SEMs from ANOVA OF Arabinoxylan Oligosaccharide (AXOS) GT43_1 RNAi lines- N1-6 and T1-6. (TIF)

S9 Table Tables of p values, means and SEMs from ANOVA OF Arabinoxylan Oligosaccharide (AXOS) Triple RNAi lines (T7, T8) and controls (C1-3). (TIF)

S10 Table Tables of p values means and SEMs from ANOVA of Arabinoxylan Oligo saccharide (AXOS) OF T1-6 v T7 + T8 (GT43_1 versus Triple RNAi wheat lines). (TIF)

S11 Table Tables of p values, means and SEMS from ANOVA of Arabinoxylan Oligo saccharide (AXOS) OF T1-6 v T7 + T8 (GT43_1 versus Triple RNAi wheat lines). (TIF)

S12 Table Tables of p values, means and SEMS from ANOVA of sugars for single GT43_1 transgenics (A) and Triples (B) and comparison of GT43_1 and triple transgenic lines (C).
(TIF)

S13 Table Marvin and Perten Seed Data. Means and standard errors of various seed traits measured in six independent GT43_1 RNAi wheat lines and their corresponding Null lines.
(TIF)

S14 Table Table of p values for seed traits analysed for six independent GT43_1 RNAi transgenic wheat lines. (TIF)

S15 Table Marvin and Perten Seed Data. Means and standard errors of various seed traits measured in two independent GT43_1+GT43_2+GT47_2 RNAi wheat lines and their corresponding control lines.
(TIF)

S16 Table Table of p values for seed traits analysed in two independent GT43_1+GT43_2+GT47_2 RNAi wheat lines and their corresponding control lines. (TIF)

S17 Table Table containing the means of each group (Single-transgenic, single-null, triple-transgenic, triple-null) for each variable, the standard errors of the means and the p values for each comparison. (TIF)
