# Supplementary material for: RNAi suppression of xylan synthase genes in wheat starchy endosperm
Source: PLoS One. 2021 Aug 19;16(8):e0256350. doi: 10.1371/journal.pone.0256350 (PMC8376096; doi:10.1371/journal.pone.0256350)
Supplement: S1 Fig — (PDF) [file pone.0256350.s001.pdf]

>TraesCS7A02G441400

GACGCGCCCGGGCTCGGCCTCCCTCCCTCACGCCTCCGTCCCCGTCCGGCACCAAACCCATCCCATCCCCTCCC
CCCTTTTTCTTCCACACCTGACCAACGATAGCCAGGCCACCGCACCCGCAGTTTCGTGCACCGCACTTCTCCACC
CACCACCTACCTCCCTCCCCCCTCCCGTGTTCCCCTCCCCCCTCAAAACCTATCGCCGCGGACCATCCACCCACC
GACGATCGATTCACGGGACTCGGAGTCGGCGTCGGCGGCGGCGGTGGCAGAGGGAGTCCGCCAGATCCGGC
CGCGGCGGCGATGATGAAGCAGCTGCTGCCGCAGAGCCAGCTGCGGCGCTCGGCGGCGGCGTCGGCGGCGC
GCTCCTCGGGGGGCGGGGTCGAGGGCGCGGGGGGCCCCGACGGCGCGGGCGCGGGCGCGGGCGGGCGGA
CCGCCTCCTCCTCCACCTTCTGGTTCCTGCTGCACGCGCTCTGCTGCCTCATCTCGCTCTTCCTCGGCTTCCGCTT
CTCCAGGCTGCTCTTCTTCCTGCTCTTCTCCAGCAGCGCGCTCTACGCGGCCACCTCCAACAACAAGTCGGCCG
TGCTCCGCGCCATCACCACCACCACCACCACCACGACCACGACGACCACCACCACCAACACCTTCACCCTCTCC
TTCGCCGCCGCGGGCAACCCGCCCCCCTCCAACCCGGACAACCTCACCGCCGCCGCGCTCGAGGAGGCCGCG
GGGAGCACGCAGAGCCACGTCGTGGTCGGCCGCCACGGGATTCGGATCCGCCCCTGGCCGCACCCCGACCCC
GTCGAGGTCATGCGGGCGCACCGGATCATGGAGCGCGTGCAGGAGGAGCAGCGCCGCTGGTACGGCGTCAG
GGAGCCGCGCCAGGTGCTCGTCGTCACCCCGACCTACTCGCGCGCCTTCCAGGCGCTCCACCTCACCGGCCTC
CTCCACTCGCTCCGCAACGTGCCCTACCCGCTCACCTGGATCGTCGTCGAGGCCGGCGGCGTCACCAACGCCA
CCGCGGCCATGCTTGCGCGCTCCAGTCTCACCTTCGTCCACGTCCCCTTCCCGGAAAAAATGCCCCTCGAATGG
GCCGACCGCCACGCCACTGAGAACCGCATGCGCCTCCACGCCCTACGGGTGATCCGGGAGAGGAAGATGGA
CGGTGTGGTTGTGTTTGCGGATGACAGCAACGTGCATAACATGGAGCTGTTCGATGAGGTGCAGAAGGTCCA
GTGGATGGCCGCGGTGTCTGTGGGTATCCTTGCGCACACCGGAACAGCAGAGCAGCCACGCCTCACCGAAGA
GGACAAGAAGAACATGCCTCTTCCAGTCCAGGGTCCTGCCTGCAACTCTTCCGGGCATTTGGCTGGGTGGCAC
ACATTCAACACGTTGCCGTTCTCTGGGAAGACCGCCACGGTGGTTGGCGAGGCAGCGCCGGTGCTACCGAAG
GGTTTGGAGTGGGCTGGGTTTGTGATGAACTCGAGAATGCTGTGGAAGGAGGCAGAGGGCAAGCCTGATTG
GGTGAAGGACCTTGATGCTGTTGGAGAGAATGGGGAGGAGATTGAAAATCCTCTTACTCTCTTGAATGACGC
GTCCTATGTTGAGCCGTTGGGGAACTGTGGAAAAAGGTCCTACTCTGGTGGCTCCGTGTTGAAGCCCGGGC
CGACAGCAAGTTTCCACAAGGGTGGGTGATTGAGCCACCTCTGGAGGTCGTTGTTCCCGCCAAGCGCACACC
ATGGCCAGAAACCACCATGGATGTTTCATCGGAGATGTTGGATGCCAAGCAAGAGCAGGAGGACAGGCAGC
TGCCAAGGACCAACAACAGGTCAGCCAGGCCCCGAAGCACCACCACGAAACGGAAGGGCGACGTCCACAAC
TGAGGCAGGATAAACAGACCATGTAATGTACATCTCACACCACCTTACACCCGCGGTATCTGCAATAGGAGGC
ATGTCCGGTCGATCTAGTGGGTATTGTGTTTATTTTTTTGTTTCCTGATGTTTTGCTCTGGTGGTAACGACGACG
GCCTCGTGCTGGGGATATGTCTTCCATAGAAGACCGTTTGTTTCGTTATCTGTAGCTCTACCTGTTGTGCTGTA
ATATAACACCGCAGTTGATGTCTCAAGAAAATGGCGTCTGAATTCTGGATGACCTATACGCATGTCAACTCTG
AAGTATGTATCTTGCGATGAATGTTTTCAAGGCACAGTTTCCCCTTTTATTGGGGCGTCGTACAACTTTTACTG
GAC

>TraesCS7A02G441400 amino acid sequence:

MMKQLLPQSQLRRSAAASAARSSGGGVEGAGGPDGAGAGAGGRTASSSTFWFLLHALCCLISLFLGFRFSRLLFFL
LFSSSALYAATSNNKSAVLRAITTTTTTTTTTTTTTNTFTLSFAAAGNPPPSNPDNLTAAALEEAAGSTQSHVVVGRH
GIRIRPWPHPDPVEVMRAHRIMERVQEEQRRWYGVREPRQVLVVTPTYSRAFQALHLTGLLHSLRNVPYPLTWIV
VEAGGVTNATAAMLARSSLTFVHVPFPEKMPLEWADRHATENRMRLHALRVIRERKMDGVVVFADDSNVHNM
ELFDEVQKVQWMAAVSVGILAHTGTAEQPRLTEEDKKNMPLPVQGPACNSSGHLAGWHTFNTLPFSGKTATVV
GEAAPVLPKGLEWAGFVMNSRMLWKEAEGKPDWVKDLDAVGENGEEIENPLTLLNDASYVEPLGNCGKKVLLW
WLRVEARADSKFPQGWVIEPPLEVVVPAKRTPWPETTMDVSSEMLDAKQEQEDRQLPRTNNRSARPRSTTTKRK
GDVHN

>TraesCS7B02G340100

ACGCCTCCGTCCCCGTCCGGCACCAAACCCATCCCATCCCCTCCCCTCCTCCTCCCTTTTCCACACTGACCAACG
ATAGCCAGGCCACCGCACCCGCAGTTTCGTGCACCGCACTTCTCCACCCACCACCTACCTCCCCCCGTGTTCCCC
TCCCCCCTCAAAACCTATCGCCGCAGACCATCCACCCACCGACGAGCGATCGCCACGGCCGATTGATCCACAG
CGCGGGGCGGTGGCGCACCAGATCCGGCCGCGGCGGCGATGATGAAGCAGCTGCTGCCGCAGAGCCAGCTG
CGGCGCTCGGCGGCGGCGTCGGCGGCGCGCTCCTCGGGGGGCGGCGTCGGGGCCGAGGGCGCGGGTGGGC
CCGACGGCGTCGGCGCGGGCGCGGGGGCGGGCGGGCGGACCGCCTCCTCCTCCACCTTCTGGTTCCTGCTCC
ACGCGTTCTGCTGCCTCATCTCGCTCTTCCTCGGCTTCCGCTTCTCCAGGCTGCTCTTCTTCCTGCTCTTCTCCAG
CAGCGCGCTCTACGCCGCCACCTCCAACAACAAGTCGGCCGTGCTCCGCGCCATCACGACAACCACGACGACG
ACCACGACGACCACAACCACGACCAACACCTTCACCCTCTCCTTCGCCGCCGCGGGCAACCCGCCCCCGTCCAA
CCCGGACAACCTCACCGCCGCCGCGCTCGAGGAGGCCGCGGGGAGCACGCAGAGCCACGTGGTGGTCGGCC
GCCACGGGATTCGGATCCGCCCCTGGCCGCACCCCGACCCCGTCGAGGTCATGCGCGCGCACCGGATCATGG
AGCGCGTGCAGGAGGAGCAGCGCCGCTGGTACGGCGTCAGGGAGGCGCGCCCGGTGCTCGTCGTCACCCCG
ACCTACTCGCGCGCCTTCCAGGCGCTCCACCTCACCGGCCTCCTCCACTCGCTCCGCAACGTGCCCTACCCGCT
CACCTGGATCGTCGTCGAGGCCGGCGGCGTCACCAACGCCACCGCCTCCATGCTTGCGCGCTCCAACCTCACC
TTCGTCCACGTTCCATTCCCGGAAAAAATGCCCCTCGAATGGGCCGACCGCCACGCCACTGAGAACCGCATGC
GCCTCCACGCCCTACGGGTGATCCGGGAGAGGAAGATGGACGGTGTAGTTGTGTTTGCCGATGACAGCAACG
TGCATAGCATGGAGCTGTTCGATGAGGTGCAGAAGGTCCAGTGGATGGCCGCAGTGTCTGTGGGTATCCTTG
CGCACACCGGAACAGCAGAGCAGCCACGCCTTACCGAAGAGGACAAGAAGAACATGCCACTTCCAGTCCAGG
GTCCTGCCTGCAACTCTTCCGGGCATTTGGCTGGATGGCACACATTCAACACGTTGCCGTTCTCTGGGAAGAC
CGCCACGGTGGTTGGCGAGGCAGCGCCTGTGCTACCGAAGGGTTTGGAGTGGGCTGGGTTTGTGATGAACTC
GAGAATGCTGTGGAAGGAGGCAGAGGGCAAGCCTGATTGGGTGAAGGATCTTGATGCTGTGGGGGAGAAT
GGGGAGGAGATTGAAAATCCTCTTACTCTCTTGAACGATGCGTCCTATGTTGAACCGTTGGGCAACTGTGGGA
AGAAGGTCCTACTTTGGTGGCTCCGTGTTGAAGCCCGGGCCGACAGCAAGTTTCCACAAGGGTGGGTGATTG
AGCCACCTTTGGAAGTTGTTGTTCCCGCCAAGCGCACACCATGGCCAGAAACCACCATGGATGTCTCATCGGA
GATGTTAGACGCCAAGCAAGAGCAGGAGGACAGGCAGCTGCCAAGGACCAACAACAGGTCAGCTCGGCCCC
GAAGCACCACCACGAAACGGAAGGGCGACGTCCACAACTGAGGCAGGATAAACAAACCATGTAATGTACATC
TCACATCACCTTACACCCGCGGTATCTGCAATAGGAGGCATGTCCAGTCGATCTAGTGGGTATTGTGTTTATTT
TTTTGTTTCCTGATGTTTTGCTCTGGTGGCAACGACGACGACGCTCTCGTGCTGGGATATGTCTTCCTAGAAGA
CCGTTTCGTTATCTGTAGCTCTACCTGTTGTGCTGTAATATAACACCGCAGTTGTTGTCTCAAGAAAATGGTGT
CTGAATTCTGGATGACCTATACGCATGTCGACTCTGAAGTATTGTGTGATCAGTGTTTTCAGG

>TraesCS7B02G340100 amino acid sequence:

MMKQLLPQSQLRRSAAASAARSSGGGVGAEGAGGPDGVGAGAGAGGRTASSSTFWFLLHAFCCLISLFLGFRFS
RLLFFLLFSSSALYAATSNNKSAVLRAITTTTTTTTTTTTTTNTFTLSFAAAGNPPPSNPDNLTAAALEEAAGSTQSHV
VVGRHGIRIRPWPHPDPVEVMRAHRIMERVQEEQRRWYGVREARPVLVVTPTYSRAFQALHLTGLLHSLRNVPY
PLTWIVVEAGGVTNATASMLARSNLTFVHVPFPEKMPLEWADRHATENRMRLHALRVIRERKMDGVVVFADDS
NVHSMELFDEVQKVQWMAAVSVGILAHTGTAEQPRLTEEDKKNMPLPVQGPACNSSGHLAGWHTFNTLPFSG
KTATVVGEAAPVLPKGLEWAGFVMNSRMLWKEAEGKPDWVKDLDAVGENGEEIENPLTLLNDASYVEPLGNCG
KKVLLWWLRVEARADSKFPQGWVIEPPLEVVVPAKRTPWPETTMDVSSEMLDAKQEQEDRQLPRTNNRSARPR
STTTKRKGDVHN

>TraesCS7D02G430700

ACACCTGACCAACGATAGCCAGGCCACCGCACCCGCAGTTTCGTGCACCGCACTTCTCCACCCACCACCTACCT
CCCCCCGTGTTCCCCTCCCCCCTCAAAACCTATCGCCGCGGACCATCTACCCACCGACGAGCGACTGCGGCCGA
TTGATCCGCAGGGCGAGGCGGTGGCGGCGGAGGGAGCCCACCAGATCCGGCCGCGGCGGCGATGATGAAG
CAGCTGCTGCCGCAGAGCCAGCTGCGGCGCTCGGCGGCGGCGTCGGCGGCGCGCTCCTCCGTCAGCGGGGT
CGAGGGCGCGGGGGGCCCCGACGGCGTCGGCGCGGGCGCCGGGGCGGGCGGGCGGACGGCCTCCTCCTCC
ACCTTCTGGTTCCTGCTGCACGCGCTCTGCTGCCTCATCTCGCTCTTCCTCGGCTTCCGCTTCTCCAGGCTCCTCT
TCTTCCTGCTCTTCTCCAGCAGCGCGCTCTACGCCGCCACCTCCAACAACAAGTCGGCCGTGCTCCGCGCCATC
ACCACCACCACCACCACCACCACCACCACGACCACCACCACCAACACCTTCACCCTCTCCTTCGCCGCCGCGGG
CAACCCGCCCCCGTCCAACCCGGACAACCTCACCGCCGCCGCGCTCGAGGAGGCCGCCGGGAGCACGCAGAG
CCACGTGGTGGTCGGCCGCCACGGGATTCGGATCCGCCCCTGGCCGCACCCCGACCCCGTCGAGGTCATGCG
CGCGCACCGGATCATGGAGCGCGTGCAGGAGGAGCAGCGCCGCTGGTACGGCGTCAGGGAGCCGCGCCAG
GTGCTCGTCGTCACCCCGACCTACTCGCGCGCCTTCCAGGCGCTCCACCTCACCGGCCTCCTCCACTCGCTCCG
CAACGTGCCCTACCCGCTCACCTGGATCGTCGTCGAGGCCGGCGGCGTCACCAACGCCACCGCCTCCATGCTC
GCGCGCTCCAGCCTCACCTTCGTCCACGTCCCCTTCCCGGAAAAAATGCCCCTCGAATGGGCCGACCGCCACG
CCACTGAGAACCGCATGCGCCTCCACGCCCTACGGGTGATCCGGGAGAGGAAGATGGACGGTGTGGTTGTGT
TTGCGGATGACAGCAACGTGCATAGCATGGAGCTGTTCGATGAGGTGCAGAAGGTCCAGTGGATGGCCGCG
GTGTCTGTGGGTATCCTTGCGCACACCGGAACAGCAGAGCAGCCACGCCTTACCGAAGAGGACAAGAAGAAC
ATGCCTCTTCCAGTCCAGGGCCCTGCCTGCAACTCTTCAGGGCATTTGGCTGGATGGCACACATTCAACACGTT
GCCGTTCTCTGGGAAGACCGCCACGGTGGTTGGCGAGGCAGCGCCGGTGCTACCGAAGGGTTTGGAGTGGG
CTGGGTTTGTGATGAACTCTAGGATGCTGTGGAAGGAGGCAGAGGGCAAGCCTGATTGGGTGAAGGACCTT
GATGCTGTGGGGGAGAATGGGGAGGAGATTGAGAATCCTCTTACTCTCTTGAATGATGCGTCCTATGTTGAG
CCGTTGGGGAACTGTGGGAAGAAGGTCCTACTGTGGTGGCTCCGTGTTGAAGCCCGGGCCGACAGCAAGTTC
CCACAAGGGTGGGTGATTGAGCCACCGTTGGAAGTTGTTGTTCCCGCCAAGCGCACACCATGGCCAGAAACC
ACCATGGATGTCTCATCGGAGATGTTAGACGCCAAGCAAGAGCAAGAGGACAGGCAGCTGCCAAGGACCAA
CAACAGGTCAGCCCGGCCCCGAAGCACCACCACGAAGCGGAAGGGCGACGTCCACAACTGAGGCAGGATAA
ACAGACCATGTAATGTACATCTCACACCACCTTACACCCGCGGTATCTGCAATAGGAGGCATGTCCAGTCGAT
CTAGTGGGTATTGTGTTTATTTTTTTGTTTCCTGATGTTTTGCTCTGGTGGTAACGACGACGGCCTCGTGCTGG
GGATATGTCTTCTGTAGAAGACCGTTTCGTTATCTGTAGCTCTACCTGTTGTGCTGTAATATAACACCGCAGTT
GATGTCTCAAGAAAATGGCGTCTGAACTTGGATGATCTATACGCATGTCAACTCTGAAGTTTGTATCGTGTGAT
GAATGTTTTCAAAGCACAGTTTCCCCTTTTATTGGGGCGTTGTACAACTTTTACTGGACTGTACCCCCTCGGTAT
AGTTGGTGATCTAAACGCTCTCATATTTCTTTACAAAGGGTATATTAGTTACTGCCTCCGTCTCACAATGTAAGA
CGTTTTCTGGGGACATTATGAGACGTTTTCTGGGCGGACAT

>TraesCS7D02G430700 amino acid sequence:

MMKQLLPQSQLRRSAAASAARSSVSGVEGAGGPDGVGAGAGAGGRTASSSTFWFLLHALCCLISLFLGFRFSRLLF
FLLFSSSALYAATSNNKSAVLRAITTTTTTTTTTTTTTNTFTLSFAAAGNPPPSNPDNLTAAALEEAAGSTQSHVVVG
RHGIRIRPWPHPDPVEVMRAHRIMERVQEEQRRWYGVREPRQVLVVTPTYSRAFQALHLTGLLHSLRNVPYPLT
WIVVEAGGVTNATASMLARSSLTFVHVPFPEKMPLEWADRHATENRMRLHALRVIRERKMDGVVVFADDSNVH
SMELFDEVQKVQWMAAVSVGILAHTGTAEQPRLTEEDKKNMPLPVQGPACNSSGHLAGWHTFNTLPFSGKTAT
VVGEAAPVLPKGLEWAGFVMNSRMLWKEAEGKPDWVKDLDAVGENGEEIENPLTLLNDASYVEPLGNCGKKVL
LWWLRVEARADSKFPQGWVIEPPLEVVVPAKRTPWPETTMDVSSEMLDAKQEQEDRQLPRTNNRSARPRSTTT
KRKGDVHN

S1 Figure: *Triticum aestivum* GT43_1 nucleotide sequences. The three GT43_1 wheat homeologues nucleotide (cDNA sequences) and amino acid sequences- TraesCS7A02G441400; TraesCS7B02G340100 and TraesCS7D02G430700 were obtained from IWGSC refseq1.1. The nucleotide sequence highlighted in green denotes the targeted sequence for the RNAi generated construct. Sequences from plants.ensembl.org.
