# Supplementary figures and images for: RNAi suppression of xylan synthase genes in wheat starchy endosperm
Source: PLoS One. 2021 Aug 19;16(8):e0256350. doi: 10.1371/journal.pone.0256350 (PMC8376096; doi:10.1371/journal.pone.0256350)

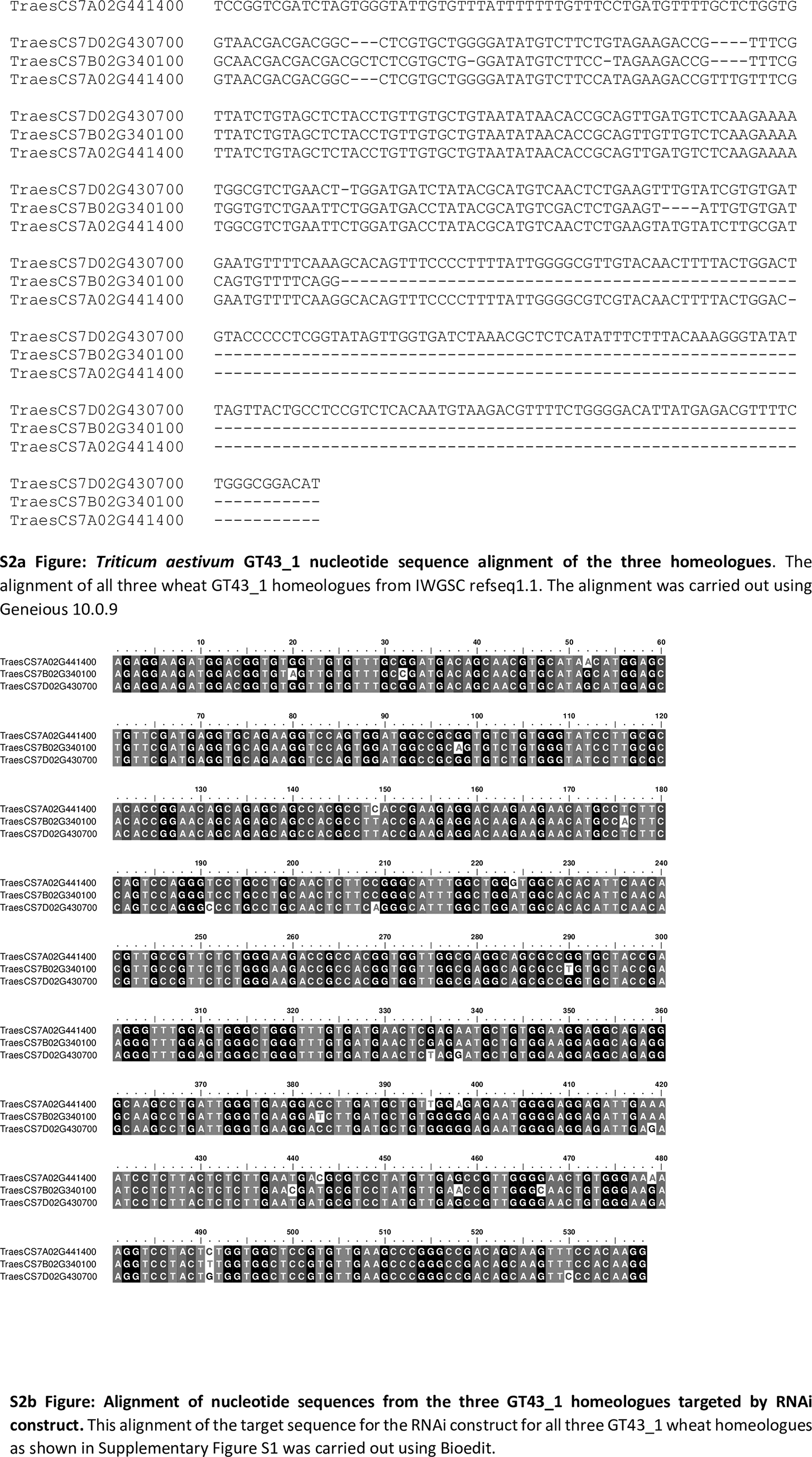

Supplement: S2 Fig — a. Triticum aestivum GT43_1 nucleotide sequence alignment of the three homeologues. b. Alignment of nucleotide sequences from the three GT43_1 homeologues targeted by RNAi construct. (TIF) [file pone.0256350.s002.tif]

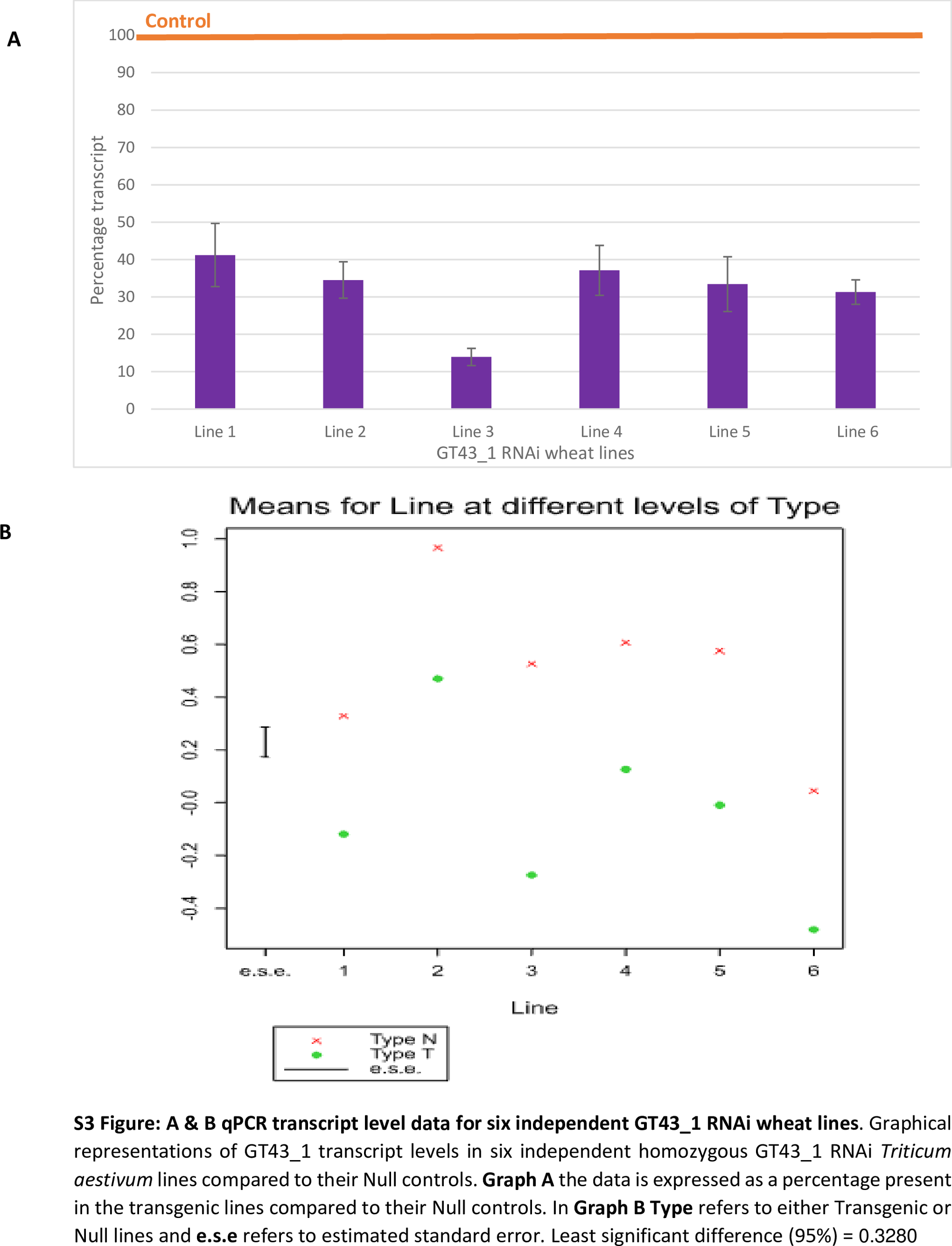

Supplement: S3 Fig — (TIF) [file pone.0256350.s003.tif]

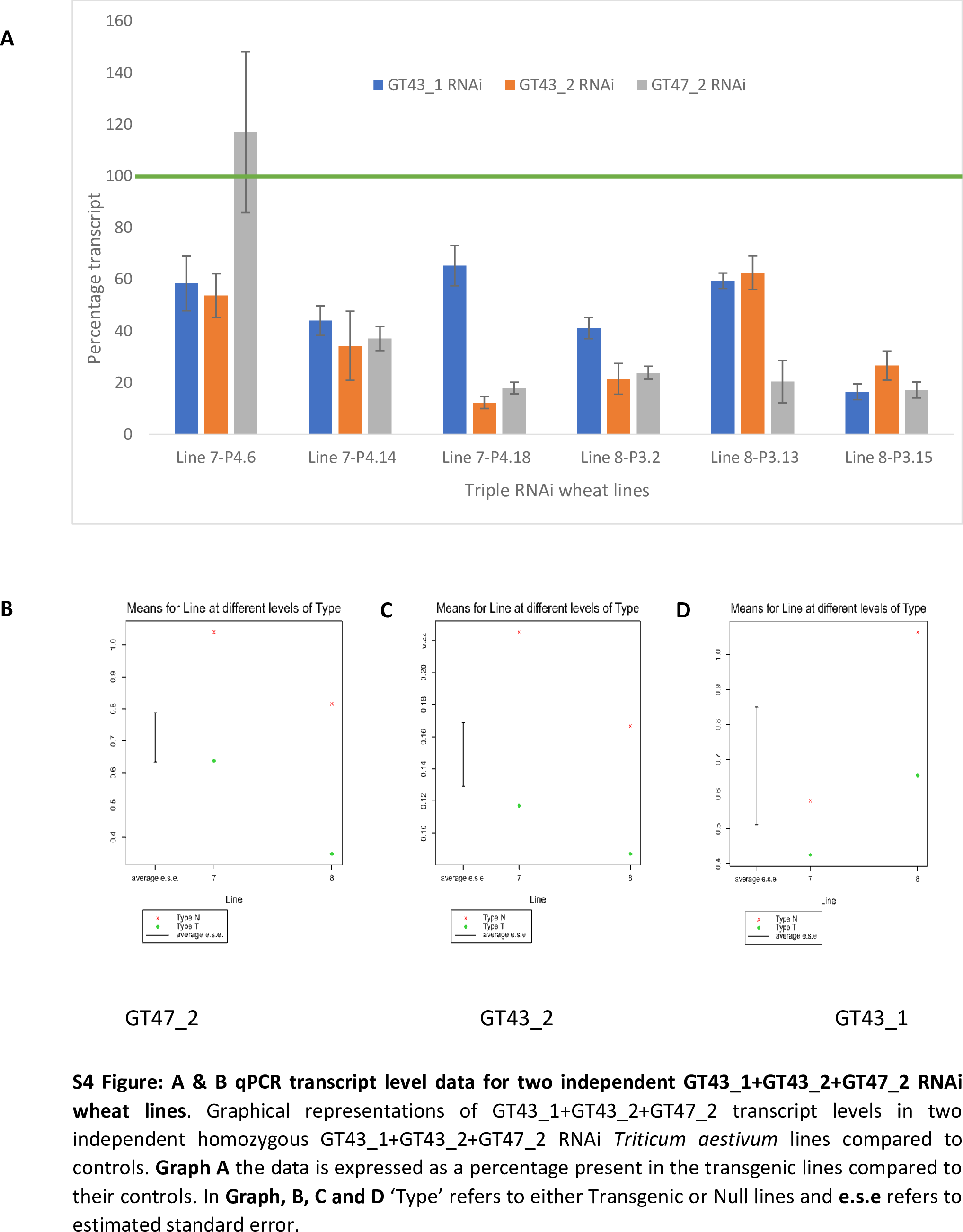

Supplement: S4 Fig — (TIF) [file pone.0256350.s004.tif]

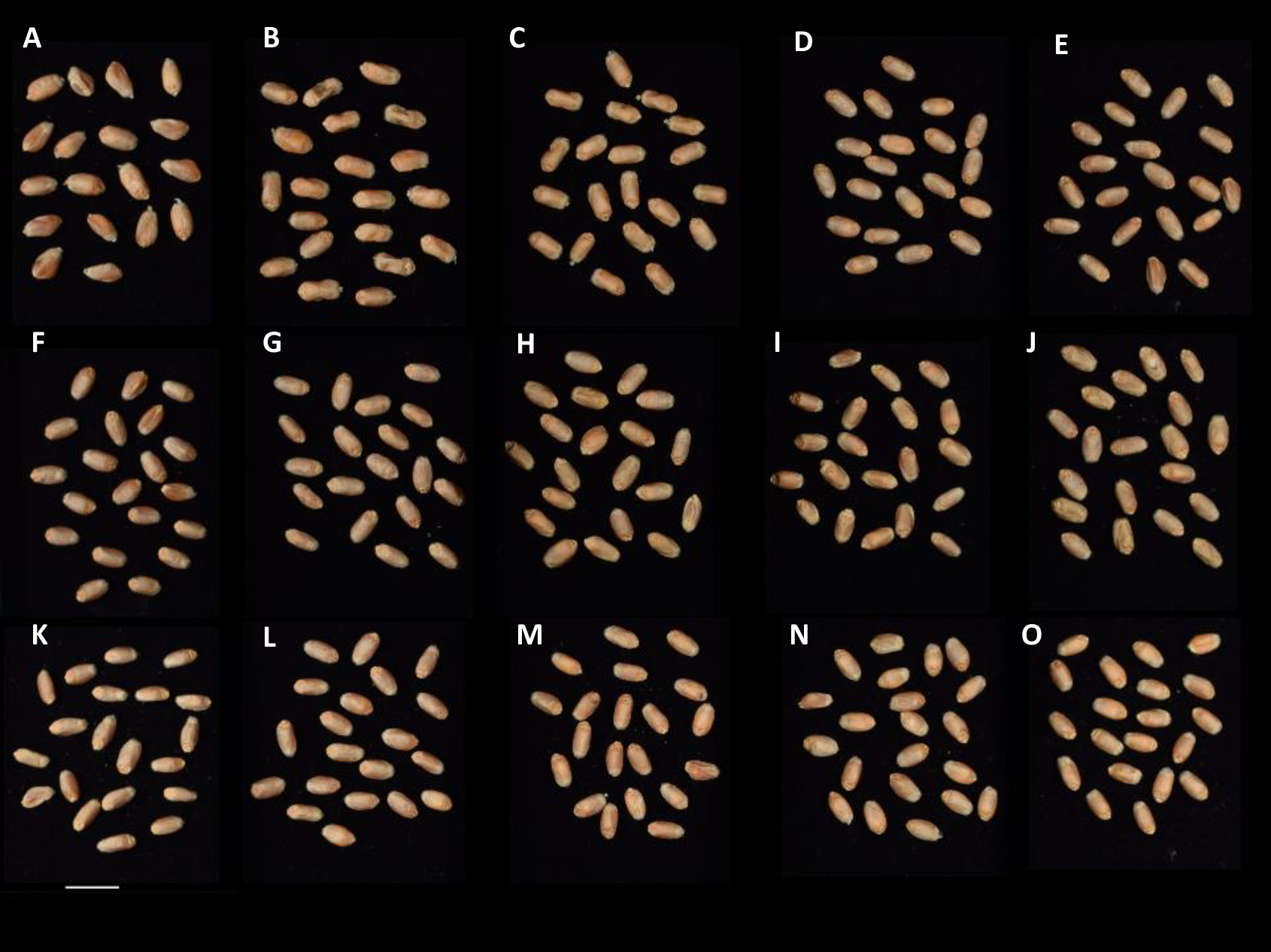

Supplement: S5 Fig — (TIF) [file pone.0256350.s005.tif]

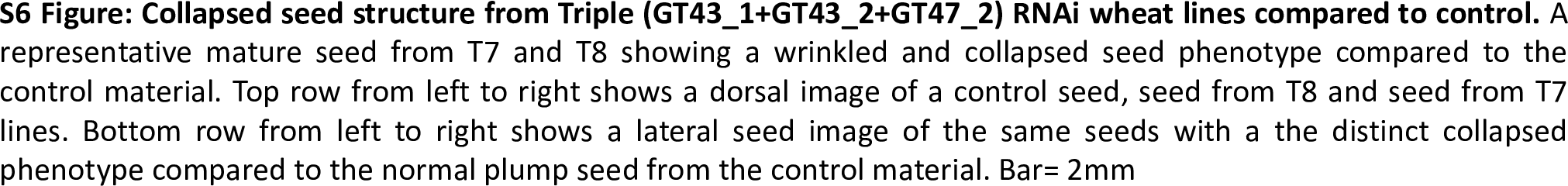

Supplement: S6 Fig — (TIF) [file pone.0256350.s006.tif]

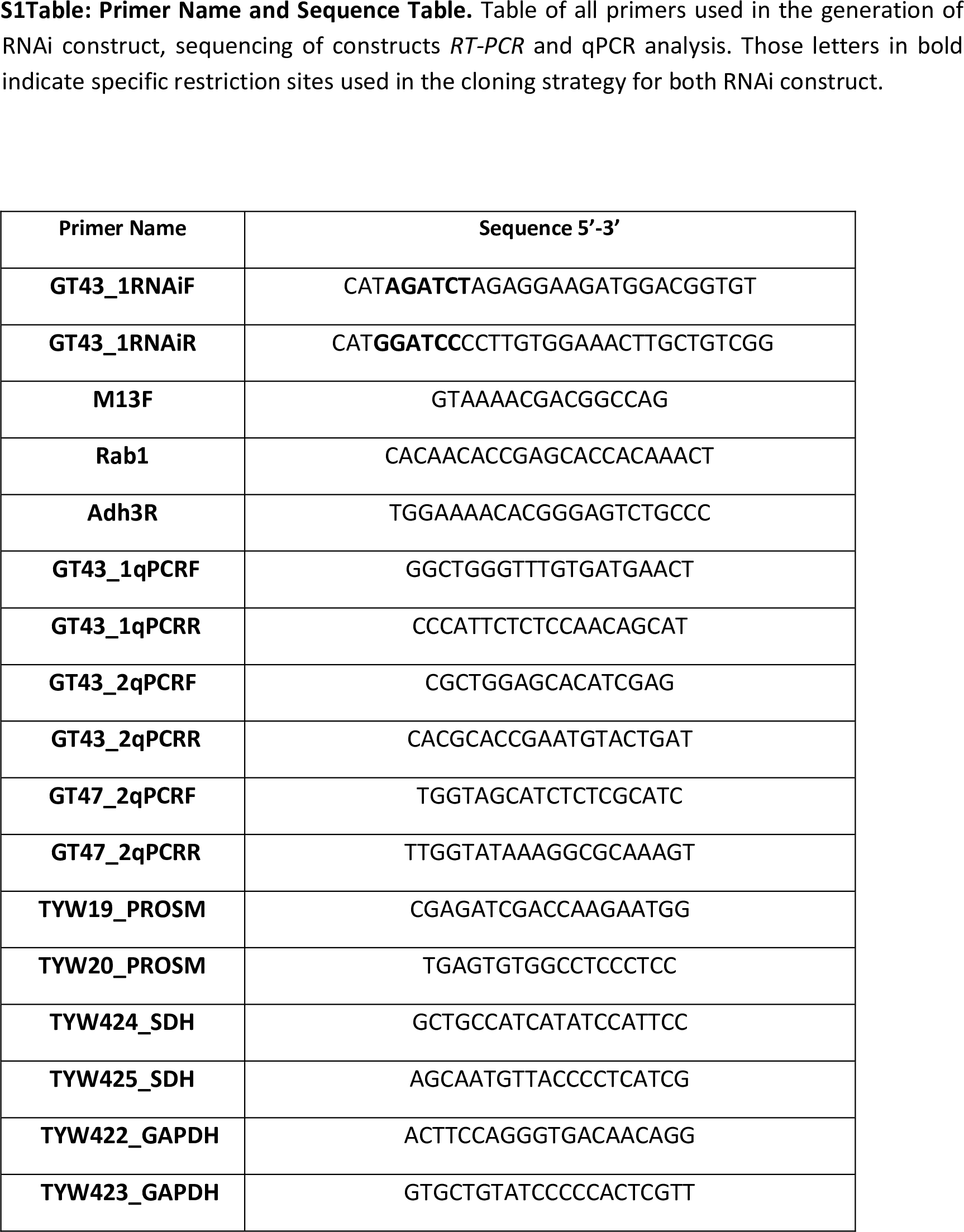

Supplement: S1 Table — (TIF) [file pone.0256350.s007.tif]

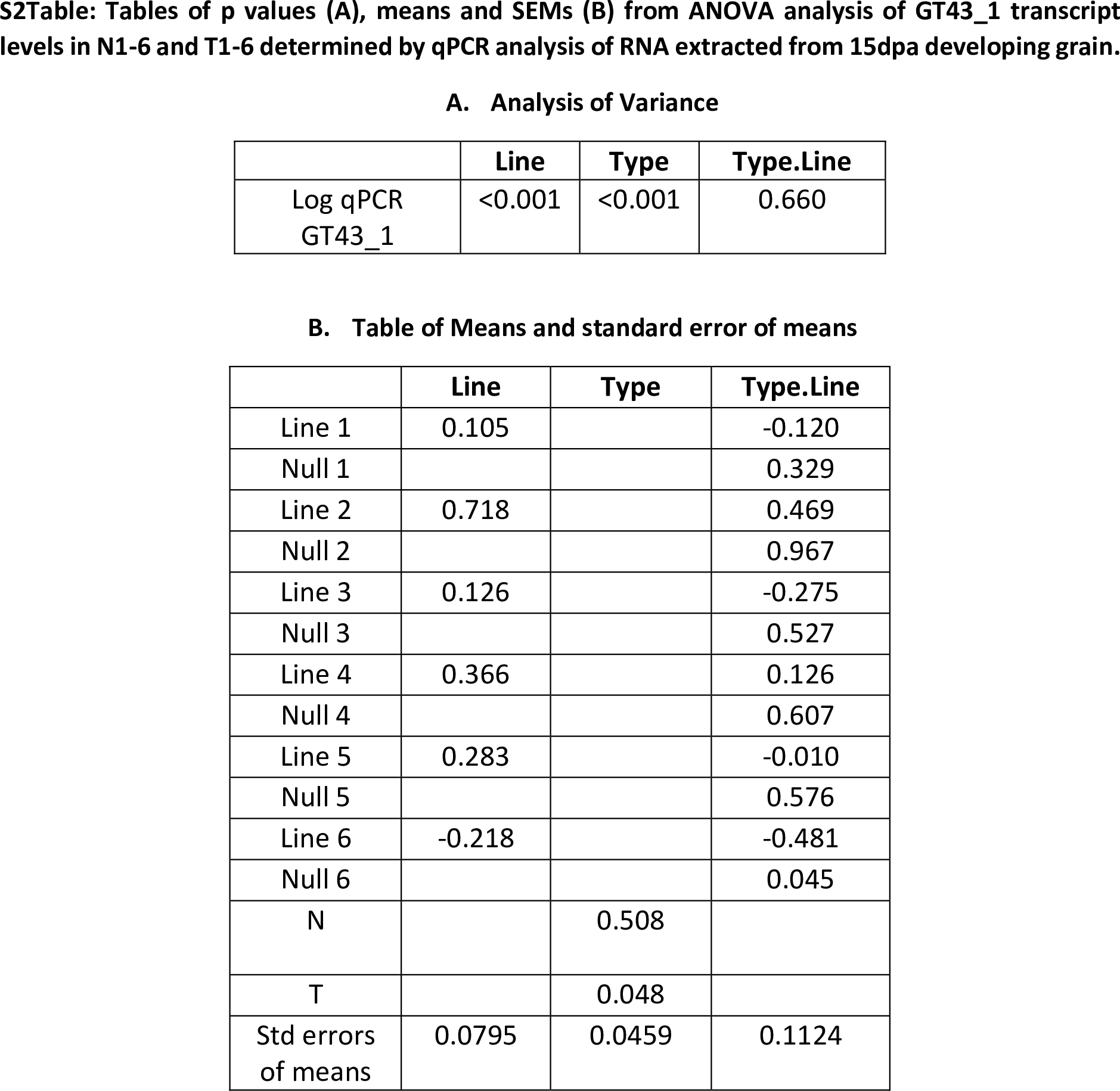

Supplement: S2 Table — Tables of p values (A), means and SEMs (B) from ANOVA analysis of GT43_1 transcript levels in N1-6 and T1-6 determined by qPCR analysis of RNA extracted from 15dpa developing grain. (TIF) [file pone.0256350.s008.tif]

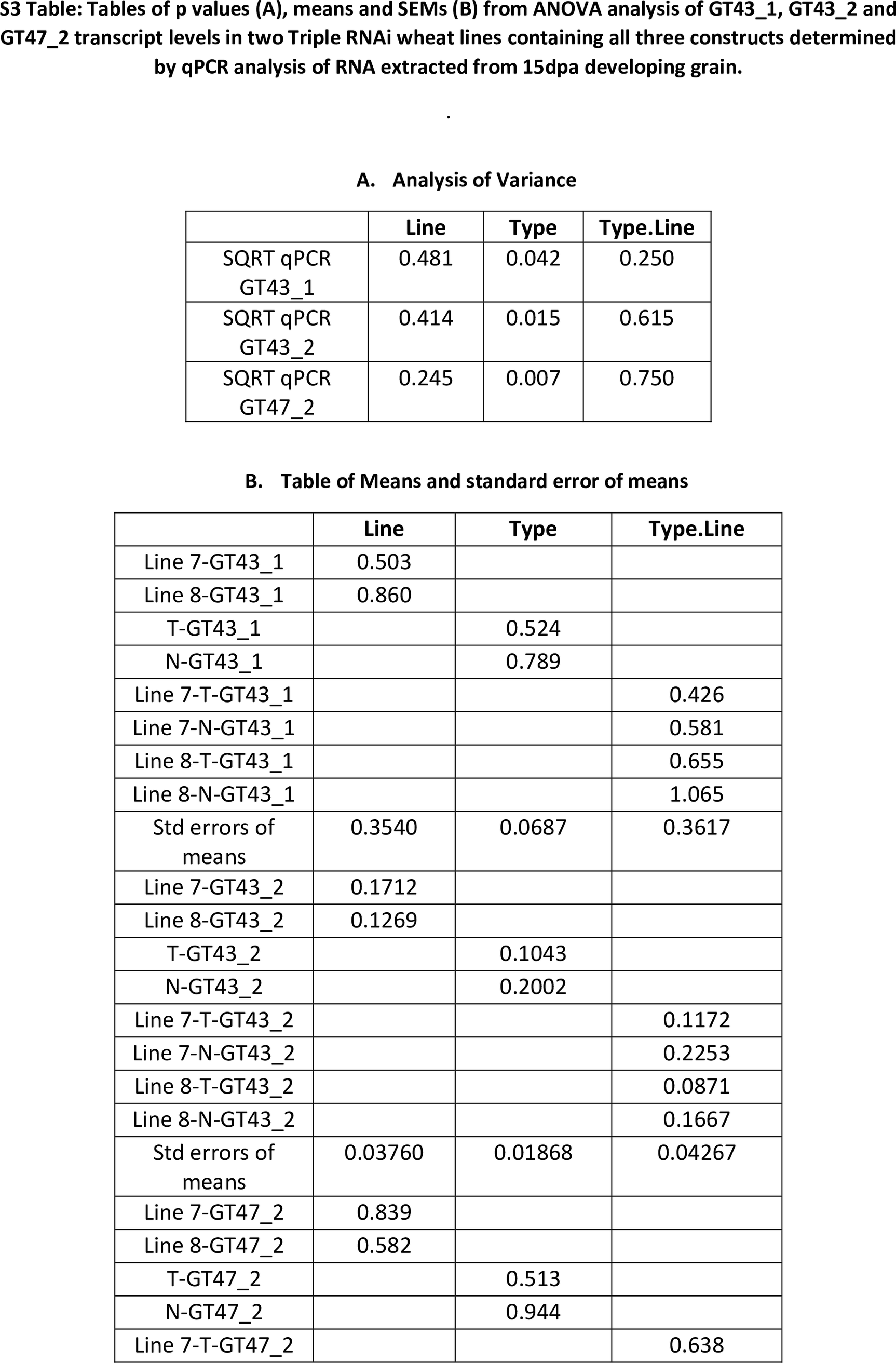

Supplement: S3 Table — Tables of p values (A), means and SEMs (B) from ANOVA analysis of GT43_1, GT43_2 and GT47_2 transcript levels in two Triple RNAi wheat lines containing all three constructs determined by qPCR analysis of RNA extracted from 15dpa developing grain. (TIF) [file pone.0256350.s009.tif]

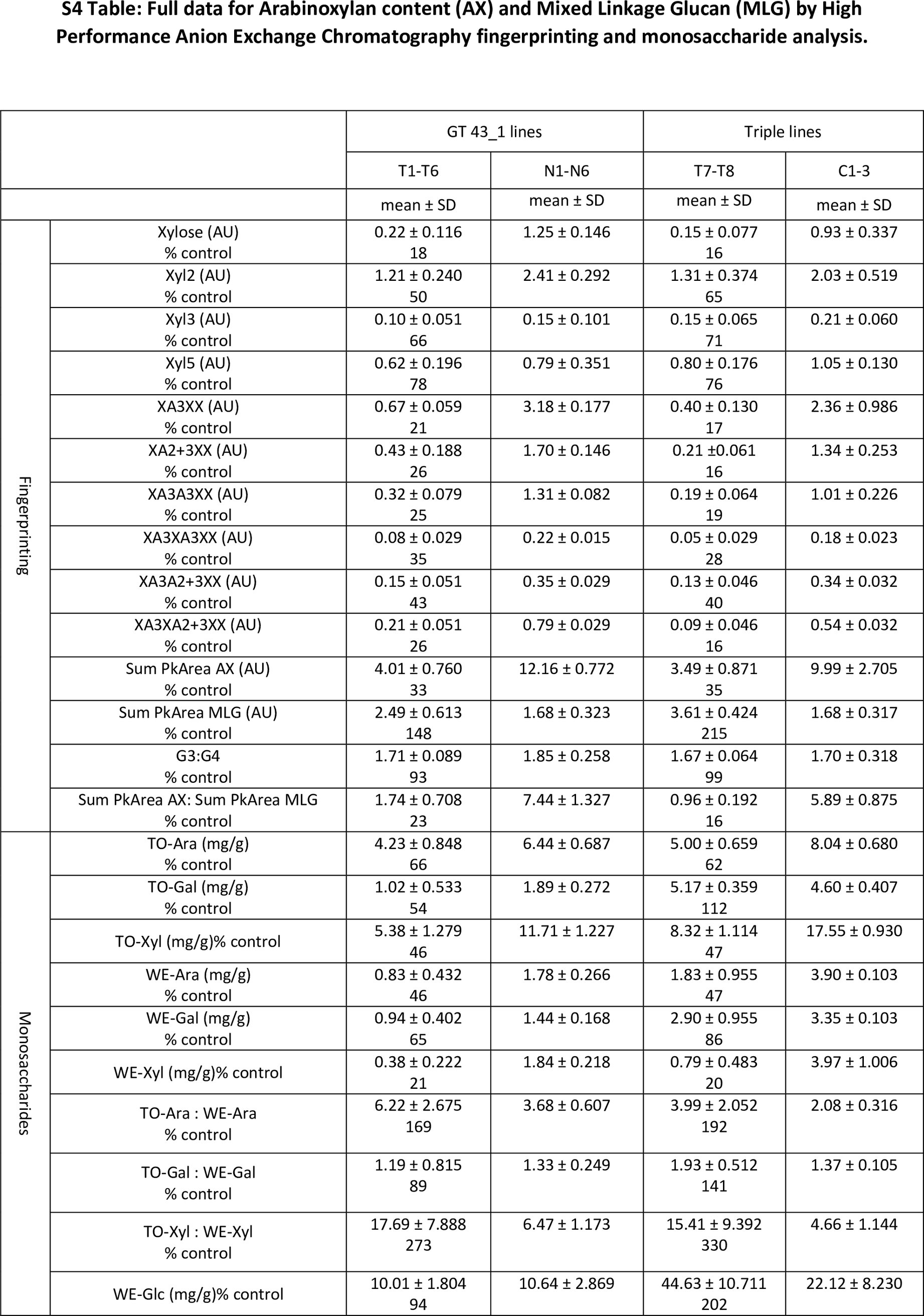

Supplement: S4 Table — (TIF) [file pone.0256350.s010.tif]

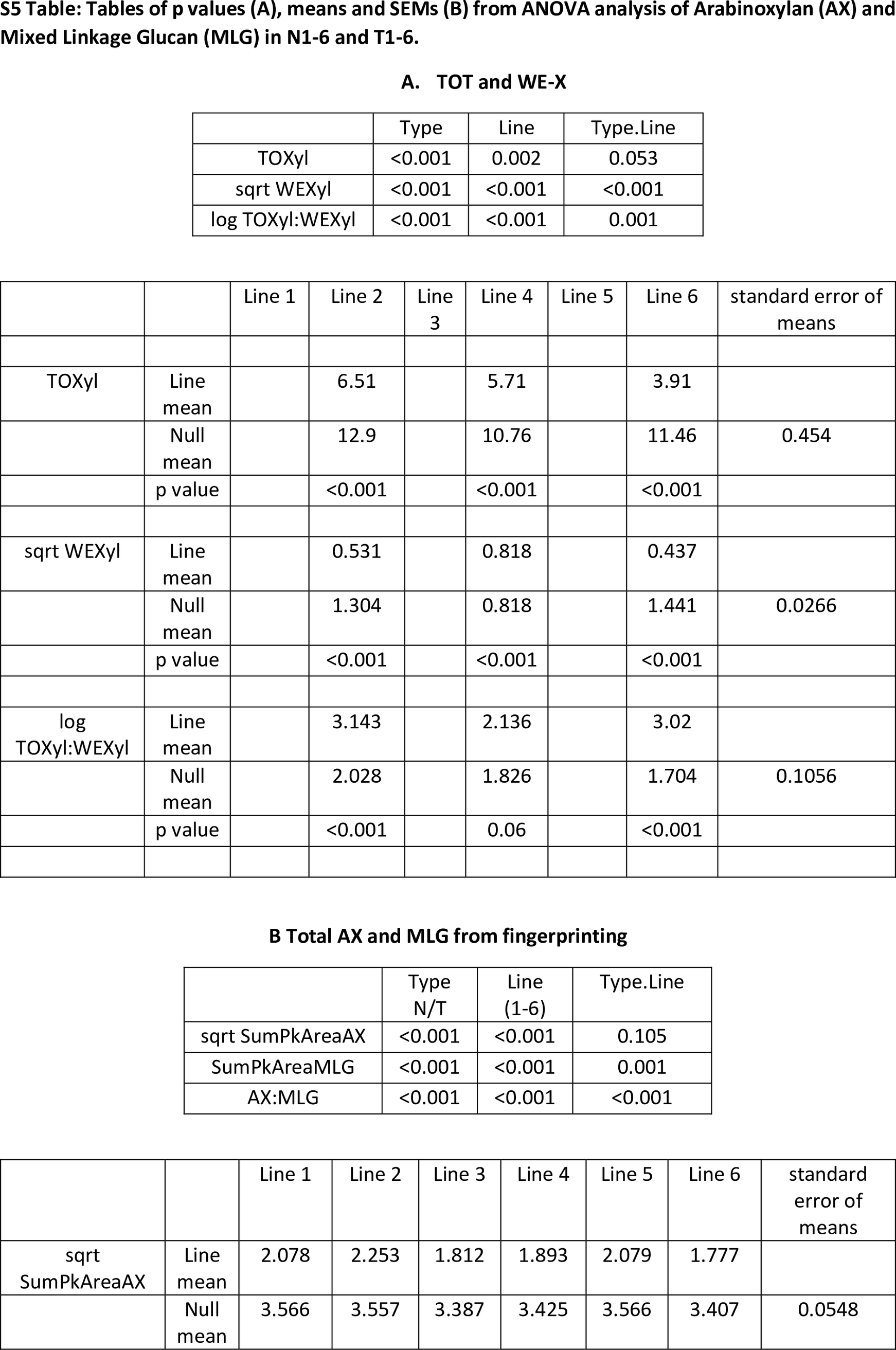

Supplement: S5 Table — Tables of p values (A), means and SEMs (B) from ANOVA analysis of Arabinoxylan (AX) and Mixed linkage Glucan (MLG) in N1-6 and T1-6. (TIF) [file pone.0256350.s011.tif]

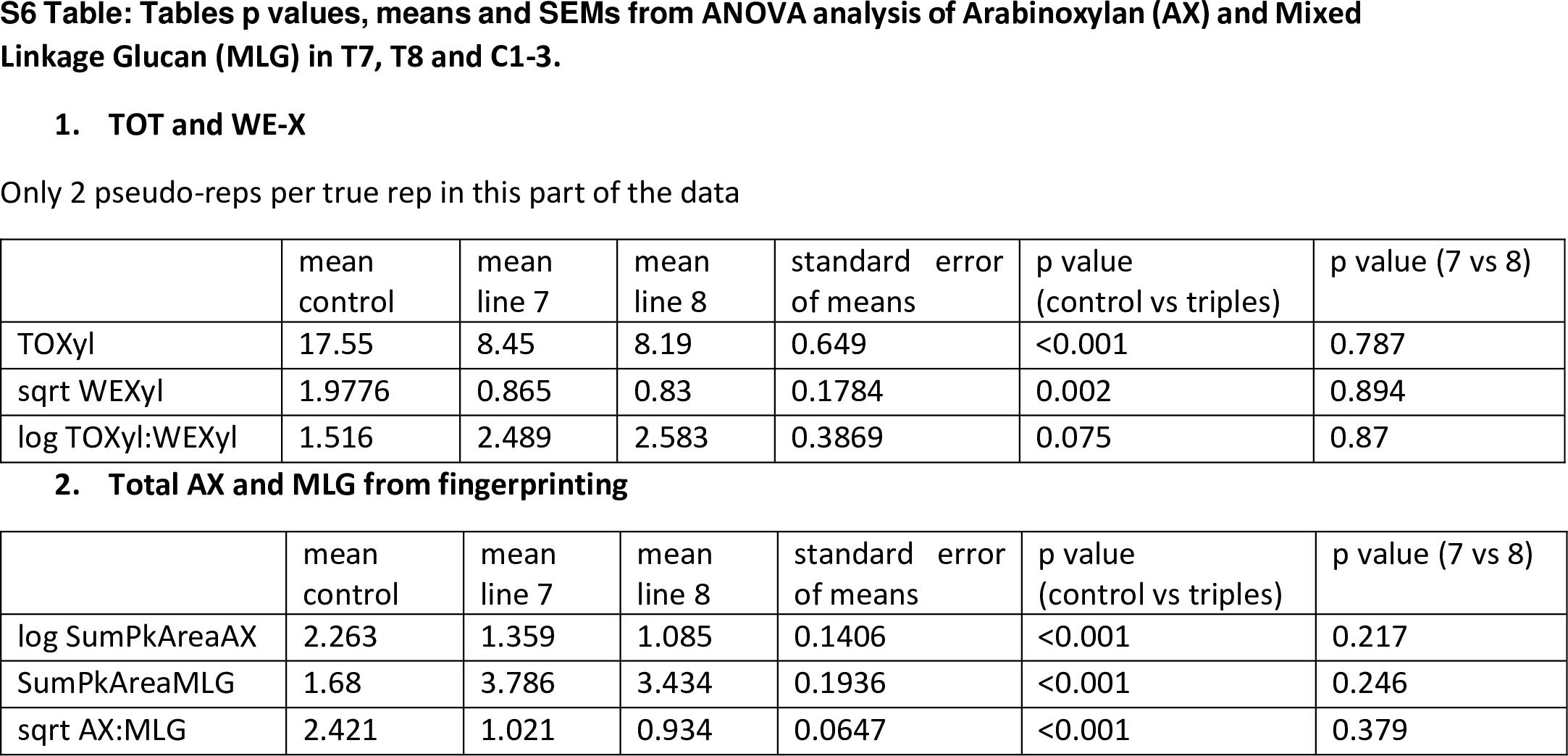

Supplement: S6 Table — (TIF) [file pone.0256350.s012.tif]

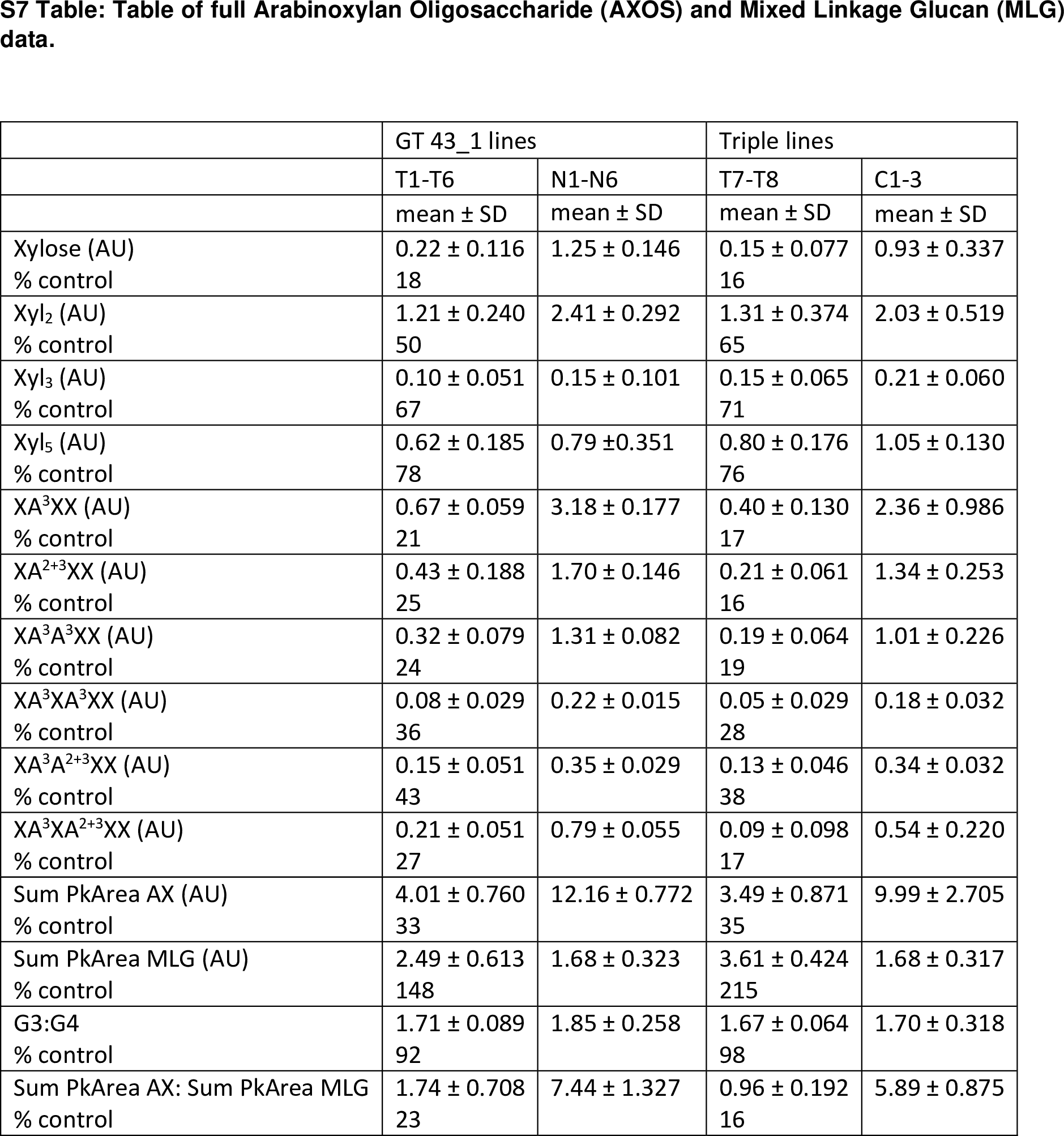

Supplement: S7 Table — (TIF) [file pone.0256350.s013.tif]

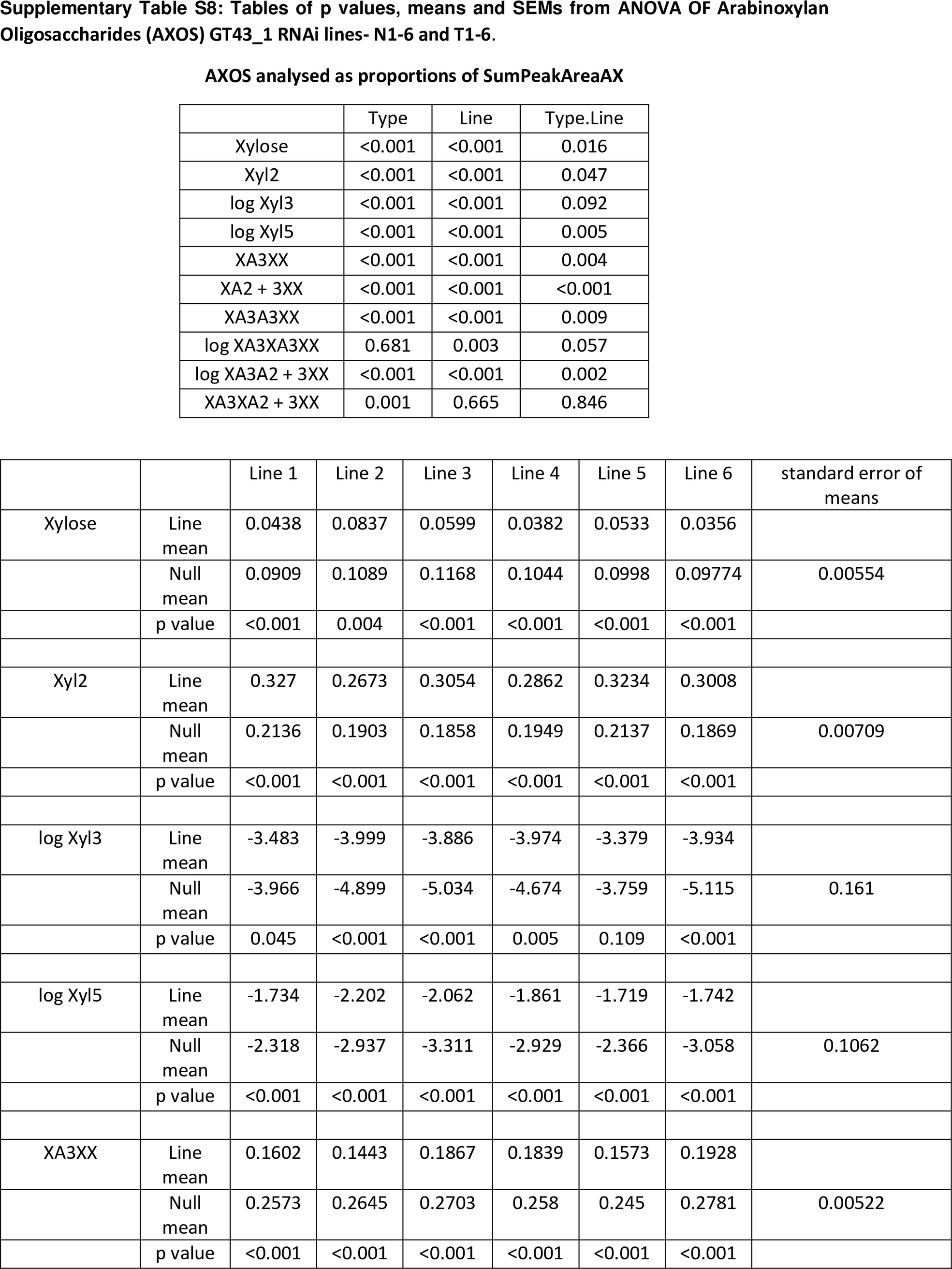

Supplement: S8 Table — (TIF) [file pone.0256350.s014.tif]

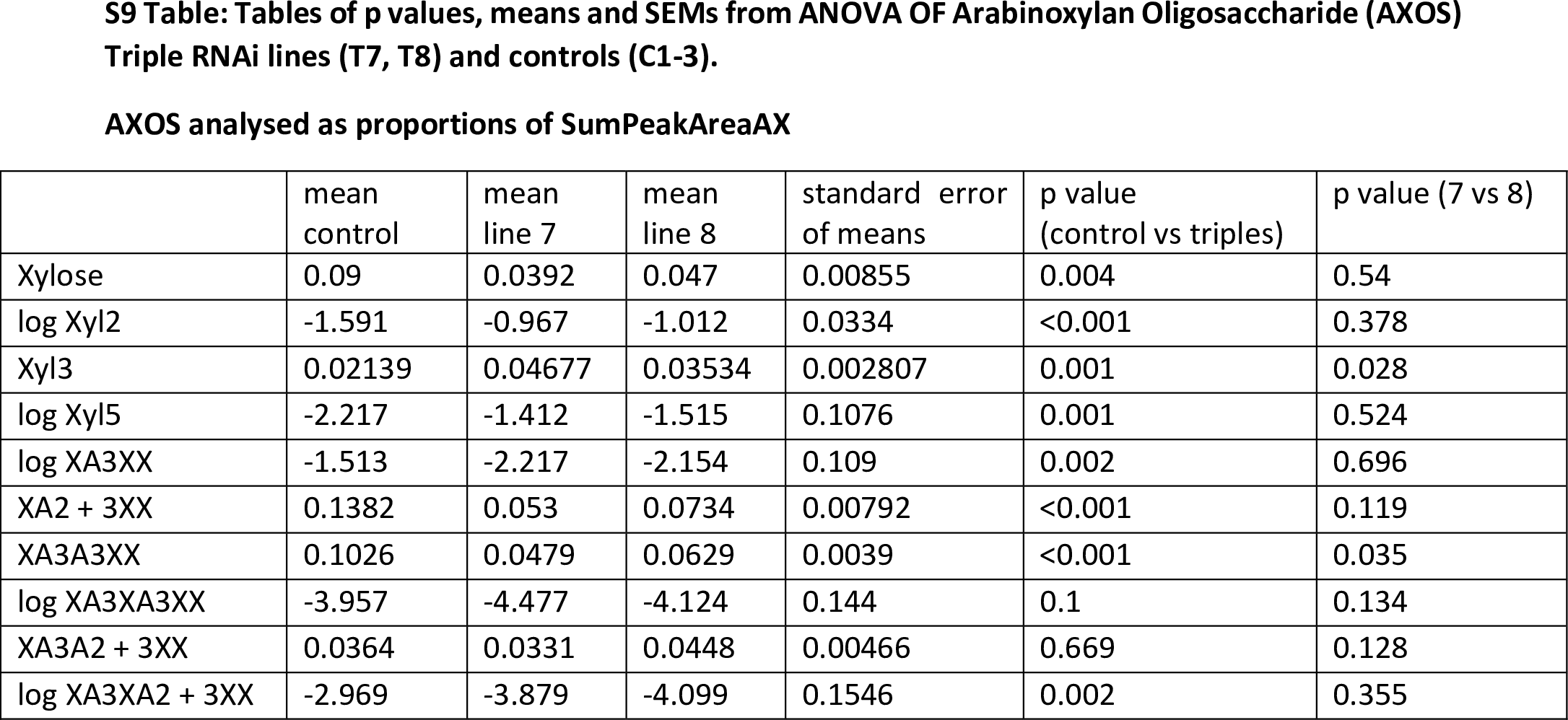

Supplement: S9 Table — (TIF) [file pone.0256350.s015.tif]

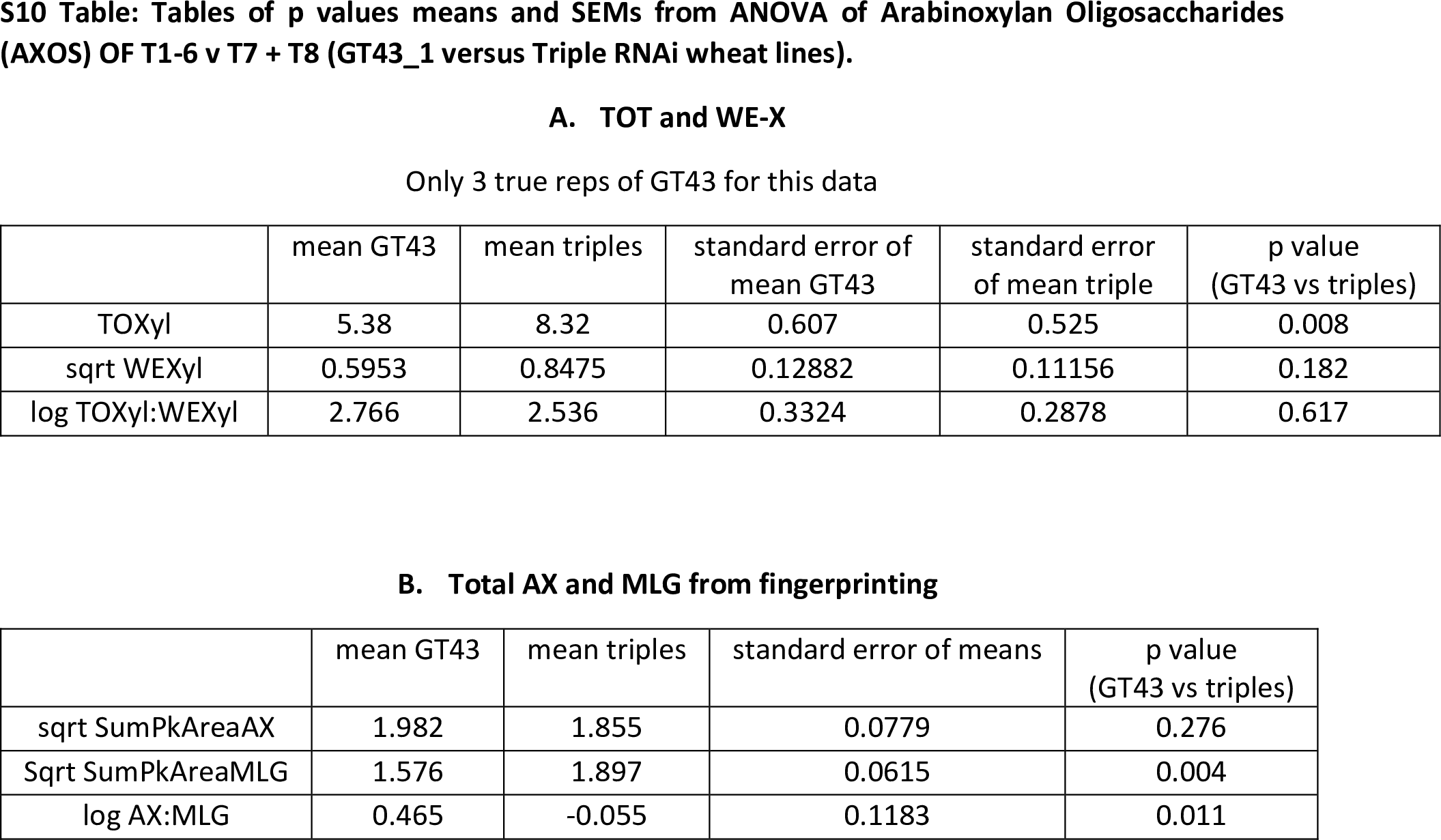

Supplement: S10 Table — (TIF) [file pone.0256350.s016.tif]

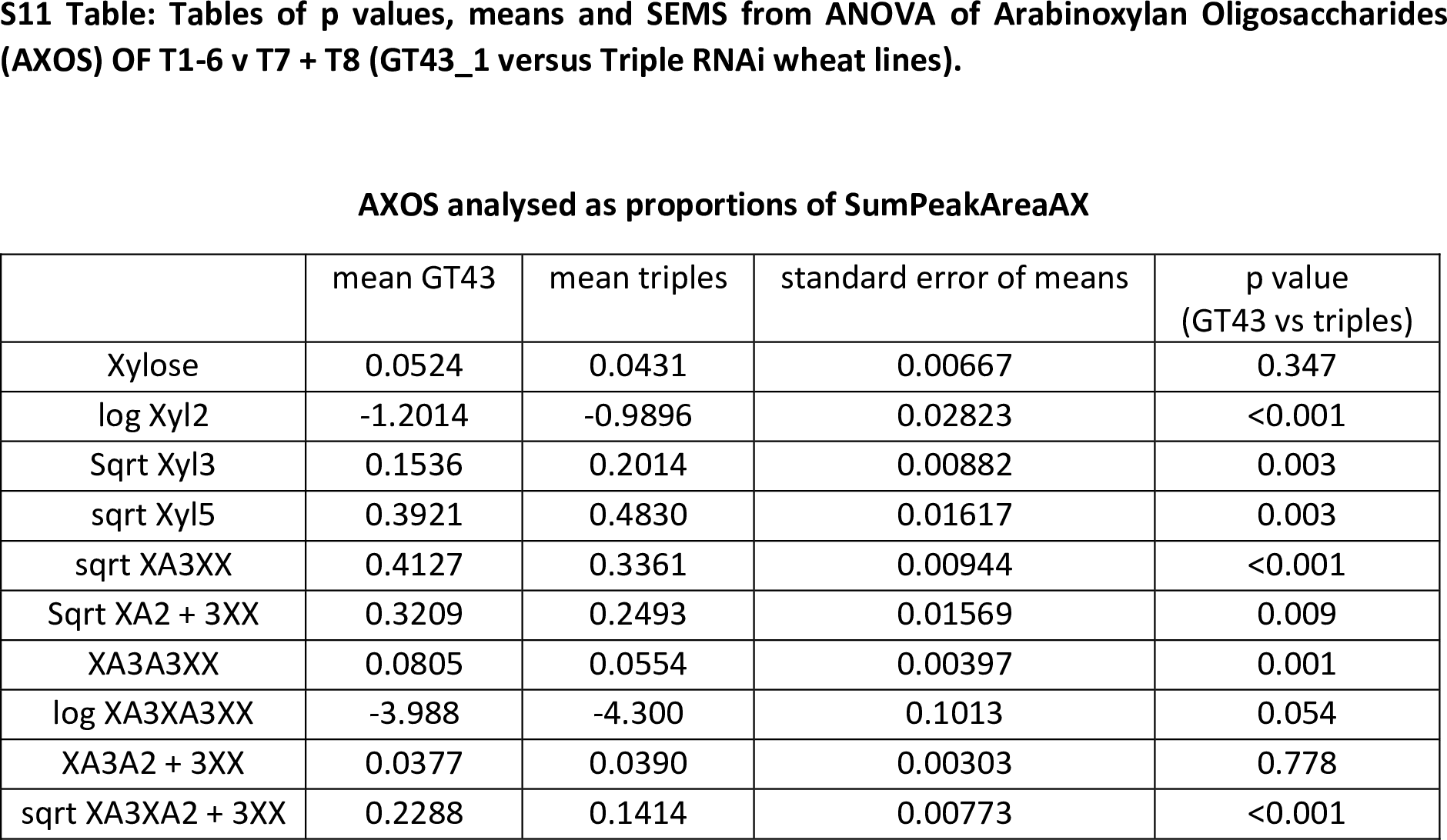

Supplement: S11 Table — (TIF) [file pone.0256350.s017.tif]

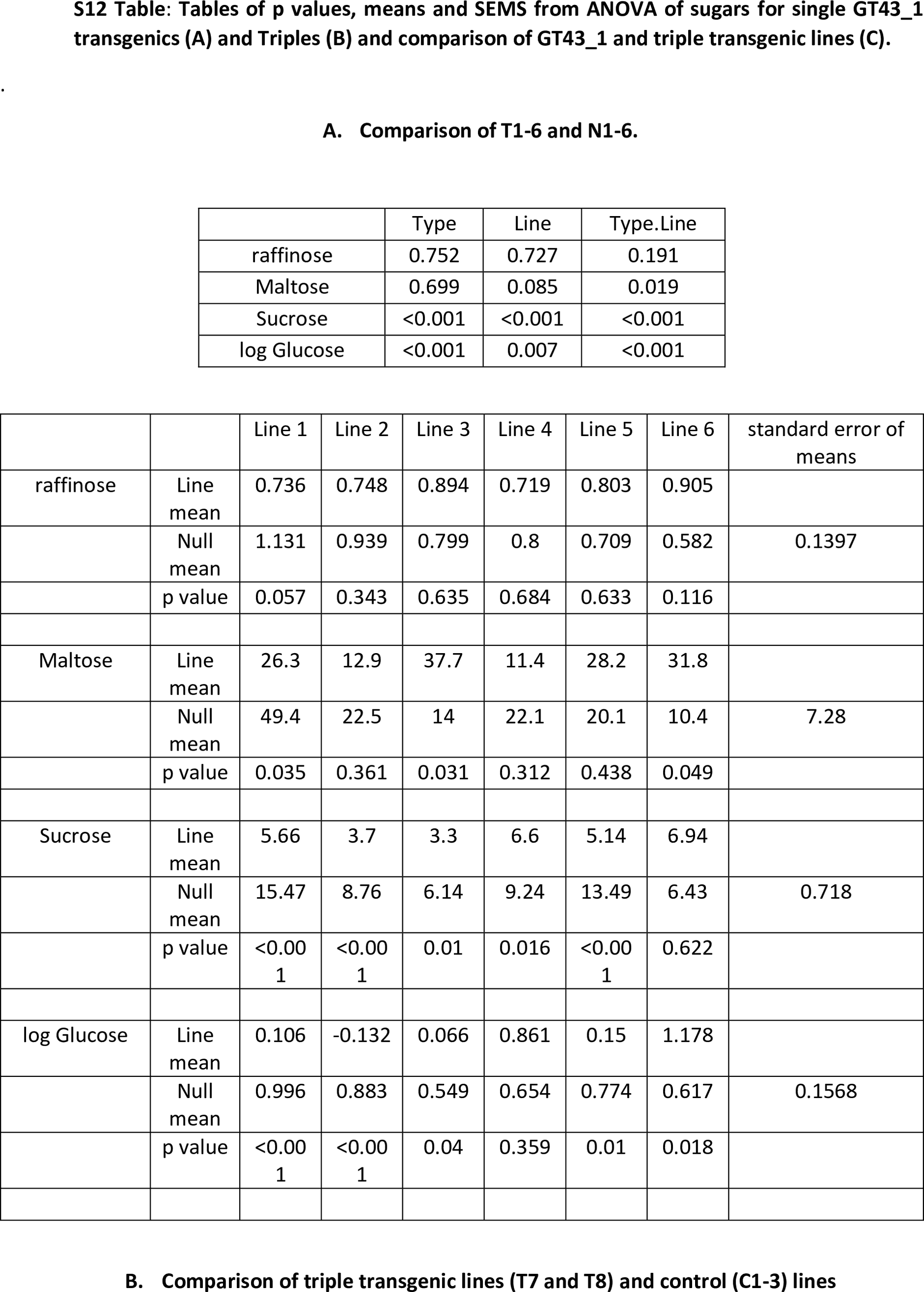

Supplement: S12 Table — Tables of p values, means and SEMS from ANOVA of sugars for single GT43_1 transgenics (A) and Triples (B) and comparison of GT43_1 and triple transgenic lines (C). (TIF) [file pone.0256350.s018.tif]

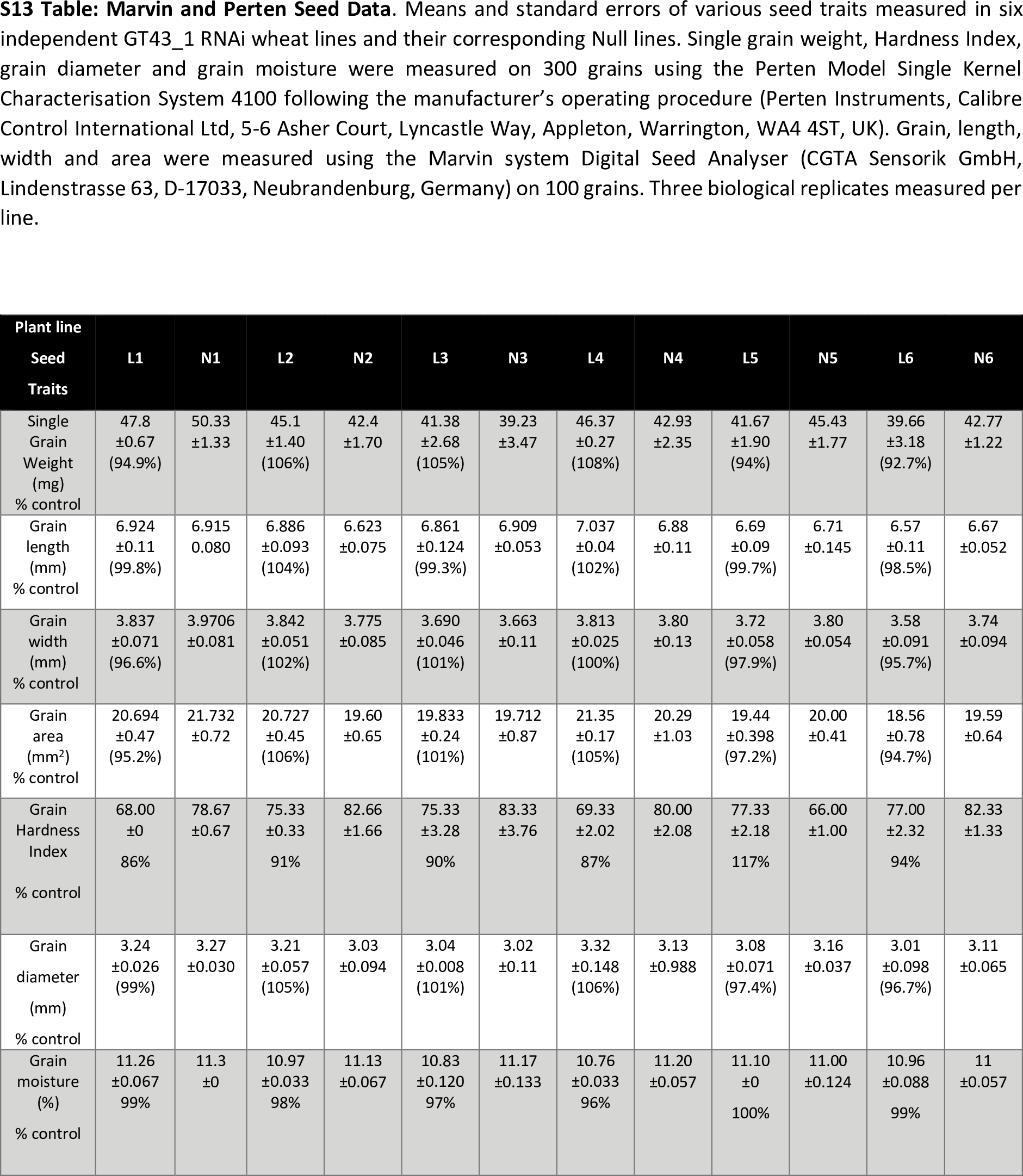

Supplement: S13 Table — Means and standard errors of various seed traits measured in six independent GT43_1 RNAi wheat lines and their corresponding Null lines. (TIF) [file pone.0256350.s019.tif]

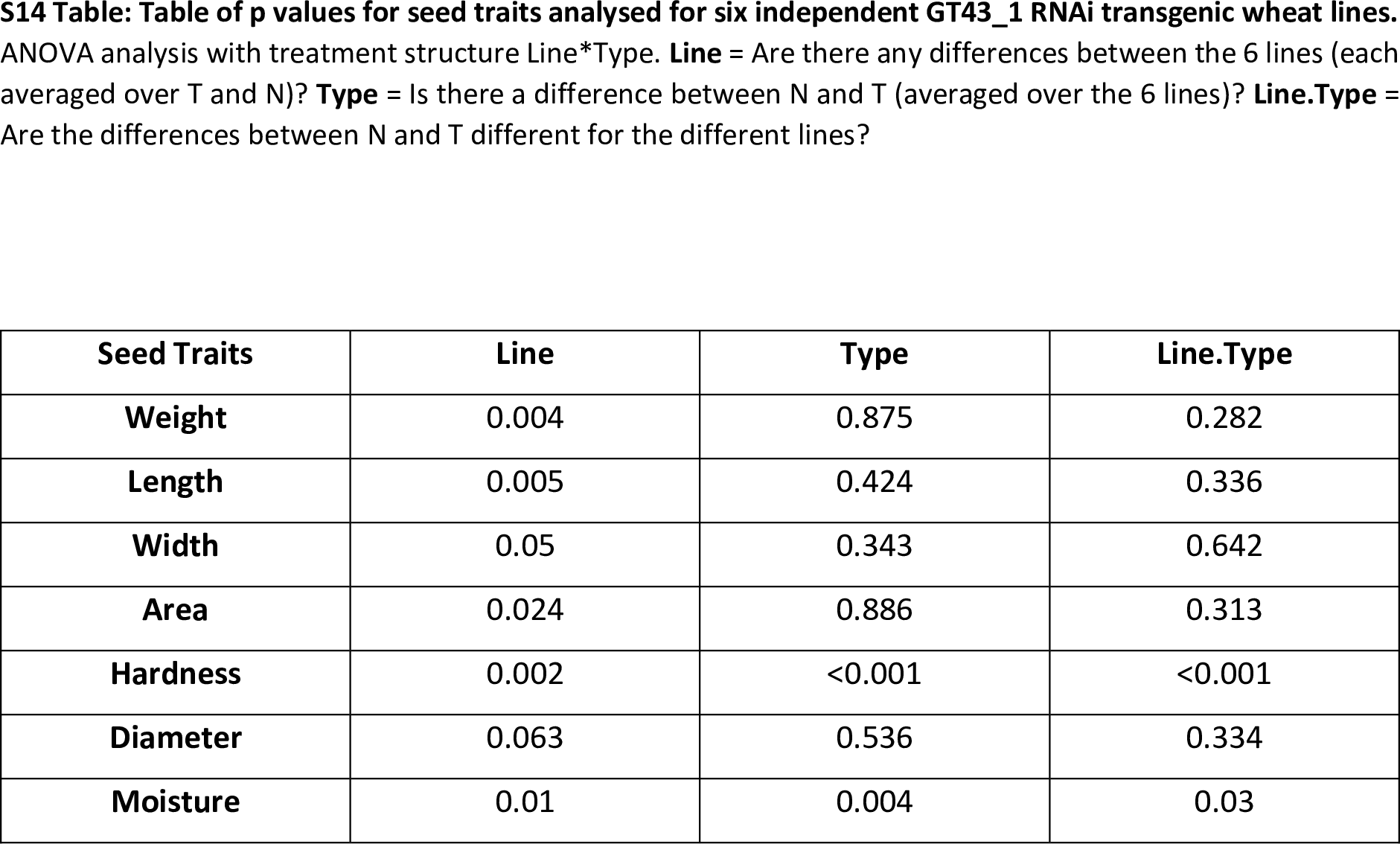

Supplement: S14 Table — (TIF) [file pone.0256350.s020.tif]

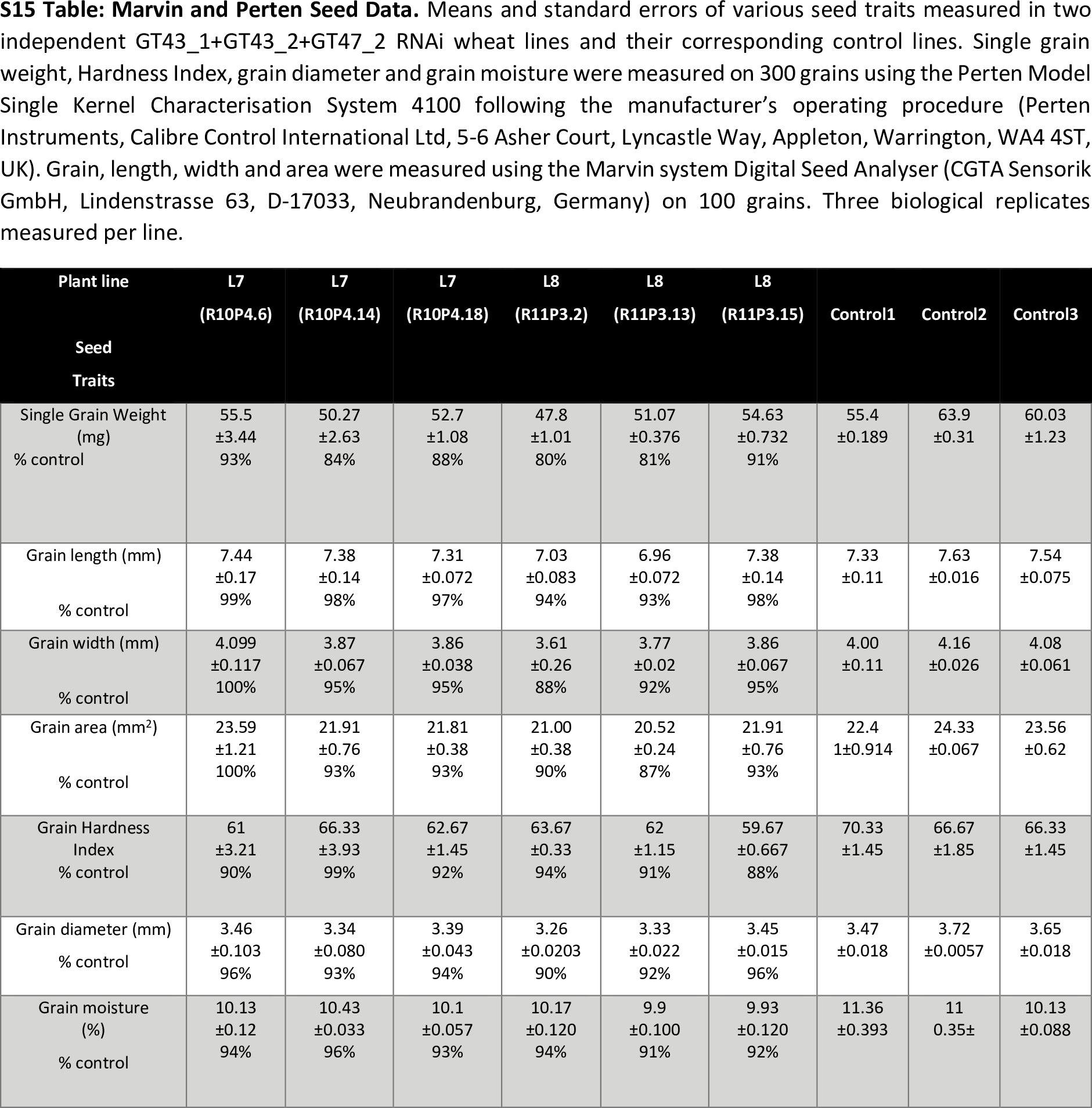

Supplement: S15 Table — Means and standard errors of various seed traits measured in two independent GT43_1+GT43_2+GT47_2 RNAi wheat lines and their corresponding control lines. (TIF) [file pone.0256350.s021.tif]

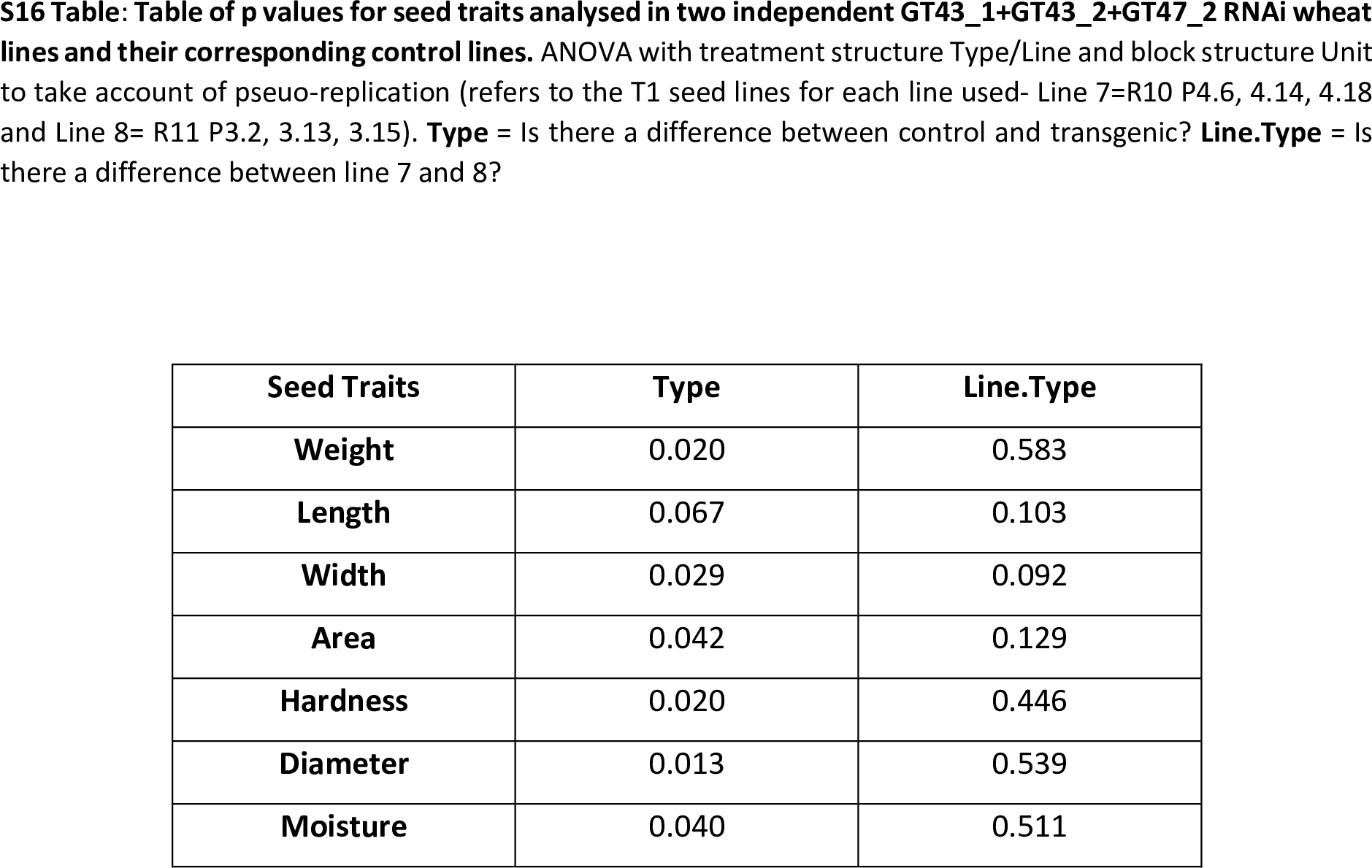

Supplement: S16 Table — (TIF) [file pone.0256350.s022.tif]

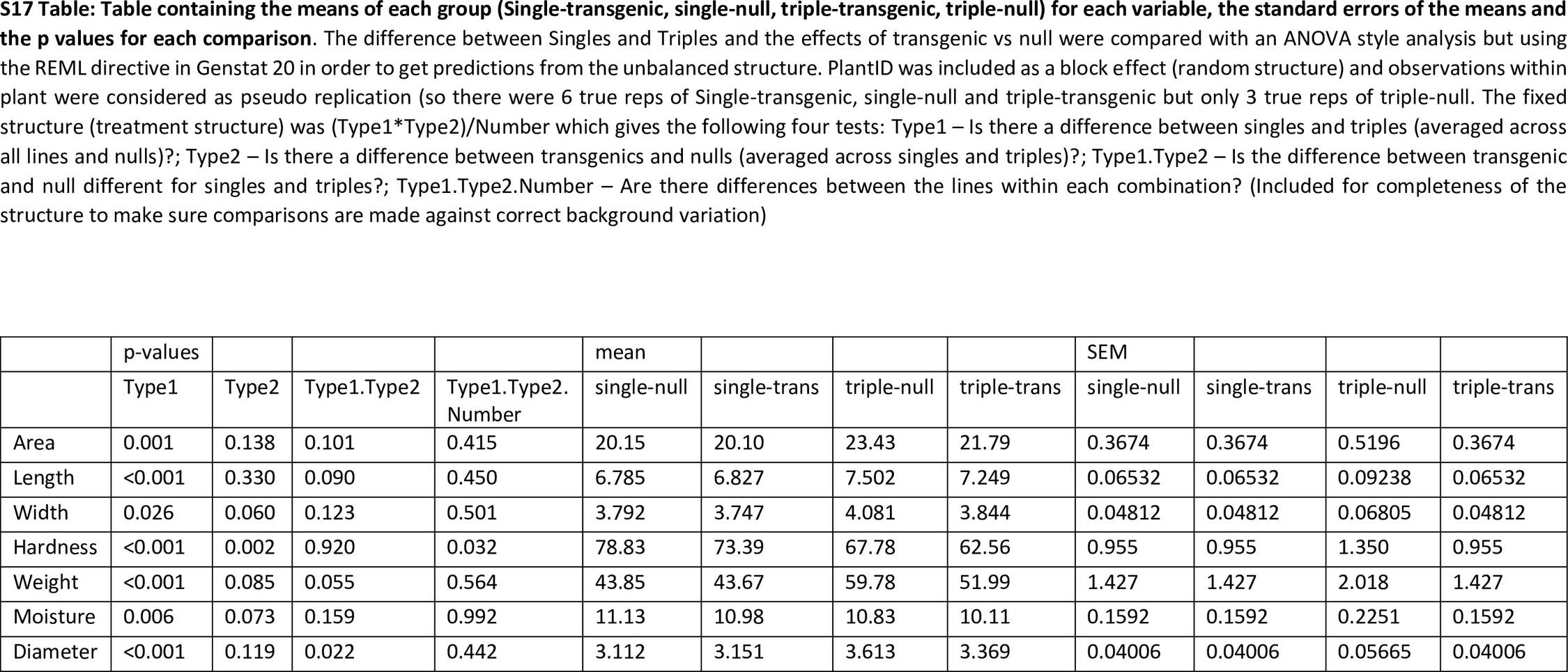

Supplement: S17 Table — (TIF) [file pone.0256350.s023.tif]
